# deepSimDEF: deep neural embeddings of gene products and gene ontology terms for functional analysis of genes

**DOI:** 10.1093/bioinformatics/btac304

**Published:** 2022-05-10

**Authors:** Ahmad Pesaranghader, Stan Matwin, Marina Sokolova, Jean-Christophe Grenier, Robert G Beiko, Julie Hussin

**Affiliations:** Montreal Heart Institute, Research Center, Montreal H1T 1C8, Canada; Faculty of Medicine, University of Montreal, Montreal H3T 1J4, Canada; Mila—Quebec Artificial Intelligence Institute, Montreal H2S 3H1, Canada; Department of Computer Science and Operations Research, University of Montreal, Montreal H3T 1J4, Canada; Faculty of Computer Science, Dalhousie University, Halifax B3H 4R2, Canada; Institute for Big Data Analytics, Dalhousie University, Halifax B3H 4R2, Canada; Institute of Computer Science, Polish Academy of Sciences, Warsaw, Poland; Institute for Big Data Analytics, Dalhousie University, Halifax B3H 4R2, Canada; Faculty of Medicine and Faculty of Engineering, University of Ottawa, Ottawa K1H 8M5, Canada; Montreal Heart Institute, Research Center, Montreal H1T 1C8, Canada; Faculty of Computer Science, Dalhousie University, Halifax B3H 4R2, Canada; Institute for Big Data Analytics, Dalhousie University, Halifax B3H 4R2, Canada; Montreal Heart Institute, Research Center, Montreal H1T 1C8, Canada; Faculty of Medicine, University of Montreal, Montreal H3T 1J4, Canada

## Abstract

**Motivation:**

There is a plethora of measures to evaluate functional similarity (FS) of genes based on their co-expression, protein–protein interactions and sequence similarity. These measures are typically derived from hand-engineered and application-specific metrics to quantify the degree of shared information between two genes using their Gene Ontology (GO) annotations.

**Results:**

We introduce deepSimDEF, a deep learning method to automatically learn FS estimation of gene pairs given a set of genes and their GO annotations. deepSimDEF’s key novelty is its ability to learn low-dimensional embedding vector representations of GO terms and gene products and then calculate FS using these learned vectors. We show that deepSimDEF can predict the FS of new genes using their annotations: it outperformed all other FS measures by >5–10% on yeast and human reference datasets on protein–protein interactions, gene co-expression and sequence homology tasks. Thus, deepSimDEF offers a powerful and adaptable deep neural architecture that can benefit a wide range of problems in genomics and proteomics, and its architecture is flexible enough to support its extension to any organism.

**Availability and implementation:**

Source code and data are available at https://github.com/ahmadpgh/deepSimDEF

**Supplementary information:**

Supplementary data are available at *Bioinformatics* online.

## 1 Introduction

In the past decades, a wide array of biological networks such as protein–protein interaction (PPI) and gene co-expression networks have come into existence. However, despite their utility, such networks are often incomplete or under-represented meaning they miss a lot of real associations and interactions. Given the fact that laboratory-based experiments are time-consuming and labor-intensive, computational algorithms have been seen as a viable solution. Many of such algorithms integrate curated ontologies, such as Gene Ontology (GO) (Ashburner *et al.*, 2000), into the original biological networks to predict missing associations. GO-based semantic similarity (SS) measures allow the comparison of GO terms by leveraging GO properties and on annotation corpora which, in turn, leads to functional similarity (FS) measurement of genes. These measures have been applied to important biological problems such as PPI prediction (Yu *et al.*, 2017), analysis of gene co-expression (Makrodimitris *et al.*, 2020), protein subcellular localization prediction (Cao *et al.*, 2018), among others, showing the vast utility of GO for the characterization of genomic and proteomic entities. For instance, Yang *et al.* (2018) proposed a new method to measure the GO-based functional similarity for miRNAs providing the community with the largest similarity matrix of human miRNAs. These similarity scores can serve as a basic feature in various prediction tasks related to miRNA functions. Schaefer and Serrano (2016) demonstrated the importance of using GO semantic similarities on pairs of genes for characterizing gene groups in cancer research, arguing that general cancer genes tend to show a higher pairwise similarity as compared to genes implicated in specific types of cancer. Kim *et al.* (2019) used GO similarity of drug-target and disease-related genes to address drug repositioning of herbal compounds. Despite these recent advances, current GO-based FS measures still depend on slow FS computation and empirical SS metric engineering. To address this inadequacy, the employment of advanced feature learning techniques offered by deep neural networks seems inevitable.

With the revival of deep neural networks around 2006 (Hinton *et al.*, 2006), deep learning methods have become prevalent in the research community. Such methods are representation learning techniques that combine multiple non-linear modules to obtain multiple levels of representation (LeCun *et al.*, 2015; Jiang *et al.*, 2017). One key advantage of deep learning is that human researchers do not design the layers of features, hence, minimal feature engineering is needed. For their promising performance, deep learning methods are increasingly being applied in the medical field including bioinformatics (Ben Ali *et al.*, 2021; Pesaranghader *et al.*, 2021a,b). For example, BioVec (Asgari and Mofrad, 2015), inspired by the Word2Vec (Mikolov *et al.*, 2013) widely used in natural language processing, is an initiative in bioinformatics to offer a solution for an unsupervised data-driven vector representation of biological sequences. It is becoming increasingly clear that these powerful approaches can help the mining of ontologies such as GO to extract meaningful insights about genes and proteins’ biological functions and interactions.

There exist two computational classes of GO-based FS measurements. *Ontology-based* methods take advantage of the GO structure by computing SS of GO terms prior to drawing on them for FS estimation. The SS measures revolve around the idea of shared Information Content (IC) (Resnik, 1995) of GO terms annotating genes. The IC-based FS measures of Resnik (1995), Lin (1998), Jiang and Conrath (1997), GraSM (Couto and Silva, 2011) and AIC (Song *et al.*, 2014) depend on these engineered SS measures. Recently, Dutta *et al.* (2018) presented a new approach (which we call clusteredGO in our evaluation) that utilized IC of the GO terms and the GO graph to do GO term clustering. In contrast to these pair-wise FS measures, group-wise FS measures such as simUI (Falcon and Gentleman, 2007), simGIC (Pesquita *et al.*, 2007) and SORA (Teng *et al.*, 2013) directly calculate FS by measuring the distance between two sets of GO term annotations. Based on Jaccard distance (Levandowsky and Winter, 1971), the group-wise measures are less computationally intensive; however, this occurs at the cost of accuracy. This process of FS estimation is executed and then reported for every GO sub-ontology separately.

*Distributional-based* FS measures are based on Firth’s idea (1957), characterizing one natural language word by its surrounding words in a given context. Our previous works (Pesaranghader *et al.*, 2014, 2016; Pesaranghader, 2019), proposing the simDEF model which compared the text definitions of two GO terms, were inspired by this notion to address several drawbacks of the ontology-based methods. Recently, Duong *et al.* (2019) introduced AicInferSentGO, a definition-based model that aimed to improve simDEF by proposing a new approach for vector representation of GO terms. Even though simDEF and AicInferSentGO demonstrated the significant advantage of vector representation of GO terms, they suffered from important shortcomings, some of which are still shared with even the recent FS measures: manual metric and feature engineering for aggregating GO-term SS scores prior to the computation of gene FS; large dimensions of GO-term vectors; and, separate consideration of each sub-ontology of GO for a biological task in hand due to uncertainty on how the downstream biological attributes from those sub-ontologies should be combined.

In this work, we introduce deepSimDEF, a paired neural network that attempts to address the above-mentioned shortcomings. Supervised deepSimDEF neural networks are designed to address the biological tasks; hence, the main output of deepSimDEF is a prediction model, where GO-term and gene-product embeddings are the by-products of the training process. Prior to training, deepSimDEF networks are typically initialized with pretrained GO-term embeddings that we compute in advance. deepSimDEF can be run in two settings: *single channel* considering sub-ontologies separately, and *multi-channel* with sub-ontologies combined. Evaluated on both yeast and human reference datasets, we demonstrate the performance of deepSimDEF against the FS measures of Resnik, Lin, Jiang and Conrath, GraSM, AIC, clusteredGO, simGIC, simDEF and AicInferSentGO (see Supplementary material S1 for their details). We also show in contrast to previous FS measures in which the estimation results hinged upon the choice of hand-engineered metrics such as Maximum (MAX), Average or Best-Match Average (BMA) to aggregate the SS scores of the two underlying GO annotation sets, a deepSimDEF network automatically learns this quantification regarding a biological application of interest, and later, measures FS of new genes and gene products.

## 2 Results

### 2.1 Experimental design and data overview

Table 1 provides an overview of the datasets prepared for the evaluation in the study (see Evaluation and validation datasets section for details). In order to include all available proteins of every experiment of the study (i.e. PPI, protein sequence homology or gene expression) in the testing phase, we used a 10-fold cross-validation approach by randomly dividing the total number of proteins into 10 non-overlapping sets. In each of 10 separate runs we held out one of those sets and all the protein pairs made of that set for testing; the rest of the protein pairs, devoid of any protein in the test set, were employed for network training. For example, in the PPI experiment on human data, out of the total number of 14 096 proteins in that experiment a test split consisted of 1410 proteins (their available pairs with PPI values); the rest of the proteins were used in training phase. After the model hyper-parameters were selected using 10% of the training set as a validation set, the final network in that fold was trained on the whole training set and then was evaluated on the held-out test set of protein pairs (for detailed hyper-parameters see Supplementary material S4). This design insured no interconnection between the pairs of gene products in the training and test data (i.e. no direct transitive inference between the protein pairs) while all protein pairs were tested. Despite the negligible variance in the results, for a solid conclusion, we repeated the permutation of proteins in every experiment 10 times and the average of all 100 runs was considered as the final result of that experiment.deepSimDEF networks learn low-dimensional vectors of GO terms and gene products, and then learn how to calculate the functional similarity of their pairs using these embedding vectors. The low-dimensional vectors of GO terms can be trained in advance by relying on natural language definitions of the GO terms and statistical measures of pointwise mutual information (PMI) and latent semantic analysis (LSA) applied to their result definition vectors (see Section 3 and Pesaranghader *et al.* (2013b, 2019)). Once GO term LSA embeddings are pretrained, a deepSimDEF network can be initialized by these vectors and then be fine-tuned on training data of biological application of interest. The network design of the deepSimDEF model also takes into account multi-channel and single-channel architectures for combined and separate consideration of GO sub-ontologies respectively.

**Table 1.** Experiments and datasets used for the evaluation of FS measures

	Yeast dataset		Human dataset		Task
	No. of gene pairs	No. of genes	No. of gene pairs	No. of genes	
PPI	50 154	4591	65 542	14 096	PPI classification
Sequence homology	26 757	3972	381 379	13 626	Sequence similarity estimation
Gene expression	37 405	2239	62 470	2361	Level of co-expression redection

GO annotations that lack curation or experimental validation have the ‘Inferred from Electronic Annotation (IEA)’ evidence code that is assumed to be the lowest confidence (Mazandu *et al.*, 2017). In the context of GO semantic similarity measures, however, the use of all evidence codes including IEA has been shown to yield improved prediction accuracy (Tian *et al.*, 2020) as IEA annotations are becoming more and more accurate. Hence, following what is common in the literature, we explored both cases of including and excluding IEAs (IEA+ and IEA–, respectively) in our FS model evaluation. Additionally, we conducted negative control experiments (see Supplementary material S1) to investigate the effect of incorrect annotations of proteins on deepSimDEF model training.

### 2.2 Semantic similarity of pretrained GO-term embeddings

Our definition-based pretraining method organizes embeddings of the GO terms within the Euclidean space. Once initializing a network, these embeddings have the potential of putting the network in a proper state for model optimization, leading to faster convergence and more accurate predictions. For three random quarry GO terms from a pool of ∼30 000 pretrained biological process (BP) terms, Table 2 shows the 5 top-most similar GO terms in terms of cosine similarity; and as expected, the returned GO terms are very close semantically. For cellular component (CC) and molecular function (MF), we observed the same behavior (see Supplementary material S2).

**Table 2.** Sense similarity results for three BP terms over pretrained embeddings

Query	GO term ID	GO term name
**Q#1**	**GO: 0072521**	**Purine-containing compound metabolic process**
1	GO: 0072523	Purine-containing compound catabolic process
2	GO: 0072527	Pyrimidine-containing compound metabolic process
3	GO: 0072529	Pyrimidine-containing compound catabolic process
4	GO: 0052803	Imidazole-containing compound metabolic process
5	GO: 0046453	Dipyrrin metabolic process
**Q #2**	**GO: 0045292**	**mRNA cis splicing, via spliceosome**
1	GO: 0000398	mRNA splicing, via spliceosome
2	GO: 0048024	Regulation of mRNA splicing, via spliceosome
3	GO: 0000380	Alternative mRNA splicing, via spliceosome
4	GO: 0090615	Mitochondrial mRNA processing
5	GO: 0000395	mRNA 5′-splice site recognition
**Q #3**	**GO: 0001116**	**Protein-DNA–RNA complex assembly**
1	GO: 0001115	Protein–DNA–RNA complex subunit organization
2	GO: 0001117	Protein–DNA–RNA complex disassembly
3	GO: 0071165	GINS complex assembly
4	GO: 0071824	Protein–DNA complex subunit organization
5	GO: 0032986	Protein–DNA complex disassembly

### 2.3 Prediction of PPIs

Protein–protein interactions play a key role in various aspects of the structural and functional organization of the cell and their knowledge provides insights into the molecular mechanisms of biological processes that lead to rational drug design (Murakami *et al.*, 2017). It has been shown that the FS values can be employed as an indicator for the plausibility of putative PPIs (Zhang and Tang, 2016).

Similar to other studies, we formulated this as a classification problem and examined how well a deepSimDEF network predicted true PPIs. We directly interpreted FS values as the classification probability of ‘Interaction’ and ‘No Interaction’. Table 3 for yeast PPI data and Table 4 for human PPI data demonstrate the results of predictions for deepSimDEF and other FS similarity measures with respect to F1-scores. Among the SS aggregation metrics used in the previous studies, MAX yielded the highest PPI prediction results, so we considered this metric in our evaluation.

**Table 3.** PPI F1-score prediction of the yeast data (FS aggregation uses MAX)

	Including IEA (%)				Excluding IEA (%)			
	ALL	BP	CC	MF	ALL	BP	CC	MF
Resnik	87.29	85.65	81.57	74.06	86.91	83.28	79.96	72.00
Lin	78.75	85.53	79.12	73.37	81.24	82.68	77.44	73.47
Jiang and Conrath	78.75	84.77	79.06	72.26	80.79	81.27	76.65	74.11
GraSM	87.55	85.33	81.35	74.16	86.83	83.26	80.08	72.16
AIC	78.39	85.71	79.13	72.99	81.18	82.40	77.70	73.73
clusteredGO	78.98	84.70	78.93	72.68	80.92	81.13	76.59	74.59
simGIC	68.22	63.31	61.56	59.27	67.84	62.52	61.22	58.62
simDEF	88.56	86.74	82.67	75.42	88.38	84.45	81.43	74.31
AicInferSentGO	88.61	86.71	82.75	75.47	88.31	84.38	81.28	74.36
deepSimDEF (random emb.)	90.05	88.88	88.08	84.77	90.07	86.71	86.45	83.57
deepSimDEF (LSA emb.)	92.78	91.57	89.58	87.64	92.99	91.68	89.35	86.69

**Table 4.** PPI F1-score prediction of the human data (FS aggregation uses MAX)

	Including IEA (%)				Excluding IEA (%)			
	ALL	BP	CC	MF	ALL	BP	CC	MF
Resnik	88.02	86.59	81.70	75.14	87.96	84.33	80.60	73.31
Lin	79.02	85.69	79.62	73.88	81.88	82.87	77.58	73.57
Jiang and Conrath	79.48	85.47	79.72	72.82	81.35	81.45	77.57	74.81
GraSM	87.97	86.58	81.83	75.12	87.59	83.53	80.38	73.02
AIC	79.78	86.05	79.37	74.10	81.52	83.50	77.66	73.69
clusteredGO	79.15	84.94	80.05	72.53	81.03	82.21	76.96	74.45
simGIC	69.33	64.19	62.34	60.66	69.16	63.56	62.47	59.32
simDEF	88.74	87.12	82.72	76.19	88.53	85.12	81.24	74.21
AicInferSentGO	88.83	87.14	82.04	75.96	88.31	84.54	81.04	74.45
deepSimDEF (random emb.)	90.69	87.63	86.71	85.13	89.91	87.12	86.51	84.54
deepSimDEF (LSA emb.)	93.68	90.60	89.12	87.80	93.12	90.19	88.26	87.38

In yeast PPI prediction, single-channel BP deepSimDEF, when initialized with LSA embeddings, achieved an F1-score improvement of ∼5% compared to the second-best methods, AicInferSentGO and simDEF (∼8.5% on average for BP, CC, and MF). With multi-channel deepSimDEF architecture, we observed a further increase of ∼1.25% compared to a single-channel deepSimDEF network of BP, which yielded the best results among the three single channels. This indicates consideration of all three sub-ontologies together increases PPI predictability. clusteredGO did not improve the results of Resnik or any other earlier IC-based FS measures, whereas the group-wise simGIC represented the worst performance among the evaluated FS measures. Comparing vector-based simDEF with AicInferSentGO, we observed similar results as expected due to their definition-based nature. Additionally, including IEA GO term annotations did not improve yeast PPI prediction.

For human PPI prediction, we observed slight improvements for almost all cases when including IEAs. The other results were consistent with our observations from the yeast PPI prediction; for example, working with single GO ontologies, BP showed better predictive power compared to CC and MF. However, when BP was combined with the other two we achieved higher F1-scores; the multi-channel deepSimDEF outperformed all FS measures including AicInferSentGO, the second-best model (93.68% versus 88.83%). Compared to random initialization of deepSimDEF we obtained a ∼3% improvement in F1-score when the embedding layer was initialized with our LSA embeddings. deepSimDEF with random weights, however, outperformed all baselines in the same category.

In order to verify that the method uses GO term vector similarities that are highly indicative of functional similarity, we further investigated if the characterization of proteins with low-level GO terms leads to better FS prediction of proteins. For this purpose, in separate experiments, we replaced the GO annotation of the proteins with their higher-level GO terms such as their parents and their grandparents, and then trained and tested the networks. For human data, when only MF was considered, the PPI prediction decreased from 87.80% to 85.36% and 82.97% for parent and grandparent considerations, respectively. This could be explained by the underspecification of given pairs with broader terms. For instance, the deepSimDEF network was able to correctly predict the interaction of P27695 and HMGB2 proteins with their original low-level GO-term annotations of GO: 0003691 (double-stranded telomeric DNA binding) and GO: 0000976 (transcription cis-regulatory region binding). Once these annotations were replaced by the broader GO terms such as GO: 0003677 (DNA binding) the networks estimated a lower probability of their interaction. We observed similar behavior for other sub-ontologies.

### 2.4 Correlation with sequence similarity

Proteins with similar sequences are usually homologous and tend to have similar functions (Cozzetto and Jones, 2017). For that reason, proteins in a newly sequenced genome are routinely annotated using the sequences of similar proteins in genomes of other species. Even though not always functional and sequence similarity tracks well (Schlicker *et al.*, 2007) information from sequence-similarity networks offers a powerful way to highlight where a potential manual or electronic GO misannotation may occur (Dessimoz and Škunca, 2017). Hence, correlation with sequence similarity data sets a benchmark for the evaluation of GO-based FS measures (Lord *et al.*, 2003).

Every gene pair in our sequence homology data is accompanied by the log-reciprocal (LRBS) and relative reciprocal (RRBS) BLAST scores indicating the level of sequence similarity of their component genes (see Evaluation and validation datasets section for the LRBS and RRBS details). Pesquita *et al.* (2008) noted that the relationship between semantically derived shared information from GO and RRBS is non-linear. Therefore, in our experiment with sequence data, the results of non-linear Spearman’s correlations were primarily considered for the evaluation of the FS measures (see Supplementary material S3 for Pearson’s correlation). Additionally, in the baseline FS measures, MAX and BMA metrics showed inconsistency in their correlation with sequence homology data as depending on the measure and sub-ontology of choice one metric worked better than the other, therefore both of these aggregation metrics were considered in our evaluation.

Table 5 shows deepSimDEF outperformed other FS measures in the correlation task with the yeast sequence homology data: on average, the initialized single-channel deepSimDEF improved the correlation results by ∼5.5% for LRBS, and >7% for RRBS. Compared to IC-based measures, distributional definition-based measures consistently showed higher accuracy. When all sub-ontologies were combined, the multi-channel deepSimDEF improved the FS results even more by at least 3% (>7% and >8% compared to the second-best measures from AicInferSentGO and simDEF in BP for LRBS and RRBS, respectively). In baseline models, in contrast to PPI experiments, the combination of sub-ontologies for the correlation computation between FS measures and sequence homology data reduced the correlation results. For deepSimDEF, however, it was otherwise indicating that the gating mechanism in the highway layer of the network (described in Section 3, Highway layer subsection) helps to learn how the shared information of two proteins should be computed (see additional results in Supplementary material S1). Similar to PPI experiments still, initialization of the networks with LSA embeddings improved the correlation results (∼6% improvement for LRBS and ∼2.5% for RRBS on average, when compared with random weight initialization).

**Table 5.** Spearman correlation of FS measures versus yeast sequence homology

		LRBS				RRBS			
		ALL	BP	CC	MF	ALL	BP	CC	MF
Resnik	MAX	0.7089	0.7269	0.5337	0.4743	0.6088	0.6378	0.5132	0.3514
	BMA	0.6066	0.5862	0.4771	0.5193	0.5236	0.5312	0.4752	0.4278
Lin	MAX	0.3831	0.6463	0.3763	0.6026	0.2512	0.4900	0.2892	0.4085
	BMA	0.5952	0.5756	0.4490	0.5866	0.4862	0.4919	0.4048	0.4478
Jiang and Conrath	MAX	0.3500	0.6504	0.2997	0.4975	0.1814	0.5030	0.2325	0.2845
	BMA	0.6190	0.6298	0.4733	0.5595	0.4958	0.5317	0.4126	0.3978
GraSM	MAX	0.3465	0.6584	0.2978	0.4895	0.1799	0.5002	0.2231	0.2911
	BMA	0.6277	0.6258	0.4659	0.5651	0.4990	0.5240	0.4154	0.3944
AIC	MAX	0.3434	0.6423	0.3094	0.5044	0.1873	0.5099	0.2275	0.2927
	BMA	0.6197	0.6215	0.4727	0.5694	0.5028	0.5348	0.4047	0.3920
clusteredGO	MAX	0.3591	0.6449	0.3027	0.4970	0.1720	0.5061	0.2277	0.2865
	BMA	0.6198	0.6282	0.4784	0.5504	0.4998	0.5237	0.4081	0.3917
simGIC		0.3140	0.6036	0.2519	0.4404	0.1237	0.4643	0.1703	0.2296
simDEF	MAX	0.4505	0.7339	0.4082	0.5946	0.2770	0.6126	0.3396	0.3845
	BMA	0.7252	0.7308	0.5661	0.6637	0.5974	0.6418	0.5079	0.4880
AicInferSentGO	MAX	0.4499	0.7314	0.4073	0.6018	0.2886	0.5996	0.3289	0.3841
	BMA	0.7252	0.7354	0.5775	0.6681	0.6049	0.6412	0.5220	0.5076
deepSimDEF (random emb.)		0.7590	0.6600	0.5918	0.7102	0.6813	0.6050	0.5438	0.6846
deepSimDEF (LSA emb.)		0.8078	0.7532	0.6077	0.7844	0.7255	0.6498	0.5409	0.6943

Table 6 shows Spearman correlation results between human LRBS and RRBS and FS measures. While the results were generally consistent with the yeast experiment findings, including that deepSimDEF outperformed all baselines with considerable margins of improvement (>7% and ∼6% compared to the second-best measure simDEF in MF for LRBS and RRBS, respectively), correlation values were less than what we achieved in the yeast experiment, which is not surprising considering the extent of the human genome and the complexity of human sequence homology data. Also, while in the yeast experiment BP yielded better results for the baseline measures, we observed that for human data it was the MF that was superior. For deepSimDEF, in both human and yeast, MF outperformed the other two sub-ontologies, yet fell short when they were combined. That is explainable as homologs are more likely to have similar functions (e.g. catalyze similar reactions) than participate in the same biological process. Also for both yeast and human sequence homology data, for Spearman’s correlation, FS measures correlated with LRBS better than RRBS.

**Table 6.** Spearman correlation of FS measures versus human sequence homology

		LRBS				RRBS			
		ALL	BP	CC	MF	ALL	BP	CC	MF
Resnik	MAX	0.4941	0.4768	0.2531	0.5834	0.4530	0.4605	0.2962	0.5222
	BMA	0.5095	0.4606	0.3288	0.5262	0.5196	0.4525	0.4212	0.5332
Lin	MAX	0.3087	0.5149	0.3500	0.3231	0.2820	0.5065	0.3374	0.2789
	BMA	0.5052	0.5081	0.3970	0.3777	0.5278	0.5035	0.4618	0.4194
Jiang and Conrath	MAX	0.2933	0.4981	0.2884	0.3734	0.2153	0.4865	0.2531	0.2730
	BMA	0.4847	0.5418	0.3995	0.3714	0.5280	0.5492	0.4506	0.3894
GraSM	MAX	0.2841	0.3787	0.2909	0.5071	0.2148	0.2713	0.2617	0.4876
	BMA	0.4884	0.3636	0.3907	0.5449	0.5311	0.3992	0.4517	0.5403
AIC	MAX	0.2931	0.3650	0.2797	0.4952	0.2146	0.2655	0.2449	0.4941
	BMA	0.4875	0.3737	0.4089	0.5514	0.5247	0.3923	0.4483	0.5563
clusteredGO	MAX	0.2944	0.3830	0.2788	0.4927	0.2101	0.2735	0.2589	0.4840
	BMA	0.4918	0.3731	0.3960	0.5330	0.5271	0.3801	0.4575	0.5561
simGIC		0.2356	0.3296	0.2307	0.4472	0.1530	0.2279	0.2013	0.4281
simDEF	MAX	0.3505	0.4383	0.3578	0.5528	0.2850	0.3374	0.3129	0.5412
	BMA	0.5541	0.4335	0.4320	0.6011	0.5823	0.4446	0.4956	0.5944
AicInferSentGO	MAX	0.3574	0.4387	0.3428	0.5515	0.2723	0.3288	0.3138	0.5430
	BMA	0.5440	0.4250	0.4383	0.6011	0.5897	0.4522	0.4956	0.5927
deepSimDEF (random emb.)		0.6437	0.5241	0.4232	0.6268	0.6425	0.5222	0.4986	0.6346
deepSimDEF (LSA emb.)		0.6723	0.5300	0.4480	0.6623	0.6514	0.5306	0.5126	0.6432

### 2.5 Correlation with gene expression

Highly correlated gene expression levels are often seen when genes are functionally related and participate in the same biological processes. Previous studies evaluated the performance of their FS measures by calculating the correlation between their estimations and gene-expression data (Bible *et al.*, 2017).

Wu *et al.* (2013) achieved poor correlations between their GO-based FS measure and gene expression from microarray data of yeast and human. They argued that the inconsistent results experienced in the previous studies indicate the correlations between GO-based FS measures and gene co-expression data are sensitive to the source of data and method of evaluation. Similar to Wang *et al.* (2007), however, we argue that this inconsistency stems from the inherent complexity of the gene expression datasets, and the fact that there exists no direct correlation between GO annotations and co-expression levels that one ideal GO-based FS measure can completely discover. We hypothesize though that deep neural networks have the potential to accommodate this non-linear complexity and discover the underlying inner dependency to the greatest degree possible.

**Fig. 1.**
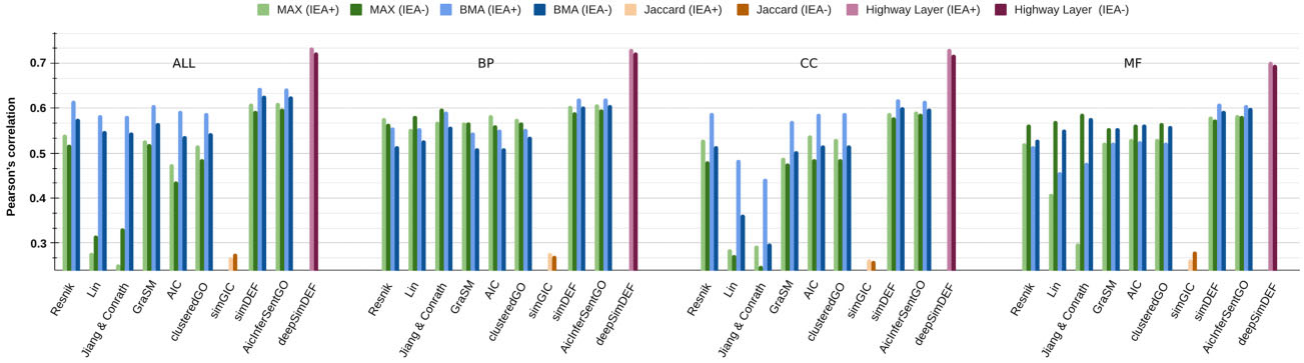
Pearson’s correlation results for the prediction of gene–gene co-expressions in yeast data

**Fig. 2.**
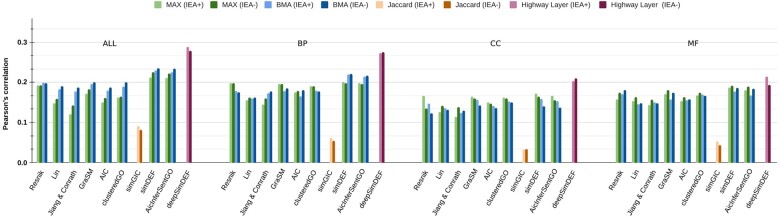
Pearson’s correlation results for the prediction of gene–gene co-expressions in human data

In our evaluation (shown in Fig. 1 for yeast experiment and Fig. 2 for human experiment), the Pearson’s correlation coefficients between the GO-based FS measures and the actual gene co-expression data were studied (see Supplementary material S3 for the exact values including Spearman’s correlation). We considered both MAX and BMA aggregation metrics in our evaluation.

We observed significantly higher correlation results for the prediction of yeast gene co-expression values relative to human values across all FS measures, which was expected given the differences in organism complexity (unicellular versus multi-cellular eukaryotes). This could also be explained by the smaller number of yeast genes leading to less complexity of co-expression patterns to discover and learn from in the training phase.

For the yeast experiment, initialized deepSimDEF was the best-performing FS measure. Single-channel deepSimDEF networks improved Pearson’s correlation with the co-expression data by >11% (average over all three GO sub-ontologies’) compared to the second-best results achieved by simDEF and AicInferSentGO (0.7238 versus 0.6146/0.6128). Regarding GO sub-ontologies, for almost all FS measures, BP showed better prediction power compared to CC and MF; for deepSimDEF this improvement over CC was much smaller. In the multi-channel deepSimDEF, we also observed a negligible increase in the Pearson correlation result over the single-channel model results in BP and CC; however, compared to MF this improvement was >3%. The multi-channel deepSimDEF outperformed simDEF, the second-best-performing measure, by ∼9% (0.7336 versus 0.6432). The inclusion of IEA also helped deepSimDEF, while for the other FS measures it was not always the case.

For the human experiment, initialized deepSimDEF was the best-performing FS measure. deepSimDEF, with the single-channel networks, improved Pearson’s correlation with the co-expression data by >6.5% compared to the second-best results achieved by simDEF (0.2442 versus 0.1774). Regarding GO sub-ontologies, similar to the yeast experiment, for almost all FS measures, BP showed better prediction power compared to CC and MF. In the multi-channel architecture also, we observed a ∼2.5% increase in Pearson correlation over the single-channel result in BP; compared to CC and MF this improvement was ∼9% and ∼11%, respectively. Additionally, the multi-channel deepSimDEF outperformed simDEF, the second-best performing measure, by ∼6% (0.2873 versus 0.2313). In contrast to the yeast experiment, however, the inclusion of IEA annotations did not always improve deepSimDEF’s results. In the baseline measures, AicInferSentGO showed comparable results to simDEF.

Similar to the PPI and sequence homology experiments, the initialization of deepSimDEF networks with our embeddings (versus random) increased the correlation results in all experiments (e.g. 0.2873 versus 0.2741 in IEA+ multi-channel deepSimDEF). deepSimDEF also outperformed other measures regarding Spearman correlation in all settings (see Supplementary material S3).

## 3 Materials and Methods

### 3.1 Experimental data

#### GO and GO annotations

In GO, GO terms are structured in three mutually exclusive sub-ontologies of *biological process* (BP), *cellular component* (CC) and *molecular function* (MF). Each GO annotation consists of an association between a gene and a GO term with an evidence code that shows how a given annotation is supported. Out of all the evidence codes, Inferred from Electronic Annotation (IEA) is the least reliable. In this study, the latest GO and the GO annotations of yeast and human genes were downloaded from the Gene Ontology website (http://www.geneontology.org/page/download-ontology (March 2021)). All genes and proteins that do not have GO annotations will be removed from our datasets, meaning that, for example, hypothetical proteins will not be investigated.

#### MEDLINE abstracts

MEDLINE (https://www.nlm.nih.gov/databases/download/pubmed_medline.html) includes >20 million citations of life sciences and biomedical articles from 1966 to the present. Combined with the GO term definitions, we employed the MEDLINE bigram list (https://www.nlm.nih.gov/databases/download/data_distrib_main.html) to build our pretrained GO-term embeddings. These pretrained GO-term embeddings initialize the first layers of the deepSimDEF networks to facilitate network optimization.

#### Evaluation and validation datasets

***Protein–* *protein* *interaction.*** From the STRING database (https://string-db.org/cgi/download.pl), we collected two lists of experimentally supported interactions for yeast and human. For negative interactions, following what is common in the literature, we independently generated random selection of pairs that were absent from the lists of positive PPIs. After removing proteins that had no GO term annotations from all three sub-ontologies (not considering IEAs), each pair of interacting proteins was labeled 1 indicating a positive interaction, or 0 offering no interaction. Our final balanced PPI datasets contained 50 154 interactions for yeast and 65 542 interactions for human proteins.

***Protein* *sequence* *homology.*** For our sequence homology datasets, we first collected the sequences of yeast and human proteins from the UniProt database in FASTA format (For the yeast: https://www.uniprot.org/proteomes/UP000002311; For human: https://www.uniprot.org/proteomes/UP000005640 (March. 2021)). We used bitscores from the Basic Local Alignment Search Tool (BLAST) algorithm Altschul *et al.* (1990) when performing an all-versus-all comparison of proteins for each organism with an expectation-value threshold of 0.1. Although this threshold is liberal, the corresponding bitscores associated with e-values near this threshold will be very low and have a minimal effect on our analysis. Since a bitscore for query and subject proteins is not symmetrical, we computed log-reciprocal BLAST score (LRBS) Eq. (1) and relative reciprocal BLAST score (RRBS) Eq. (2) to express the general sequence similarity of yeast protein pairs. After computation of LRBS and RRBS, for the yeast organism, we had a dataset of 26 757 protein pairs along with their LRBS and RRBS sequence similarity scores. This number for human protein pairs was 381 379. All proteins in these final datasets had GO annotations from the BP, CC and MF sub-ontologies (non-IEA and non-ND annotations).

(1) $$\mathrm{LRBS}(A,B)=\text{log}\;\left(\frac{\mathrm{Bitscore}(A,B)+\mathrm{Bitscore}(B,A)}{2}\right)$$

 

(2) $$\mathrm{RRBS}(A,B)=\text{log}\;\left(\frac{\mathrm{Bitscore}(A,B)+\mathrm{Bitscore}(B,A)}{\mathrm{Bitscore}(A,A)+\mathrm{Bitscore}(B,B)}\right)$$

***Gene* *expression-Yeast***. From the microarray gene expression data from Eisen *et al.* (1998), our gene expression dataset was built by integrating their data constructed for 2465 yeast genes under 79 biological conditions (four experiments on cell cycle, sporulation, temperature shock and diauxic shift processes). We first computed the absolute Pearson correlation of all possible gene–gene pairs based on the expression values regardless of their sign as we focused on the strength of co-expression, and then applied Fisher’s *z* transformation to these results to convert them into normally distributed variables suitable for parametric statistical testing. After removing those genes that had no GO annotations, all the genes in the result set had their own GO annotations from all three sub-ontologies (without considering IEAs and NDs). Due to sub-sampling from this large set, since the final dataset could be easily over-represented with co-expressed gene pairs that have small correlations, we considered a binning strategy with which the absolute Pearson correlations of pairs could fall into one of five non-overlapping bins of size 0.2 between 0 and 1. In our random sub-sampling process, we allowed only an equal number of gene pairs within the bins. The final dataset contained 37 405 gene–gene pairs along with the transformed Pearson’s correlation of their expressions.

***Gene* *expression-Human* (*Homo sapiens*).** In order to demonstrate the utility of the approach in human genomics, we used data coming from the Genotype-Tissue Expression (GTEx) portal (https://gtexportal.org/), which is a comprehensive public resource to study tissue-specific human gene expression and regulation Ardlie *et al.* (2015). Although their samples are collected from 54 non-diseased tissue sites across nearly 1000 individuals, as a proof of concept we limited our experiment to whole blood tissue gene expression available for 754 individuals. A total of 2361 genes had complete GO annotations and were retained for further analysis. The absolute Pearson correlation value was calculated between their pairs to infer the strength of the relationship; this was followed by Fisher’s *z* transformation. Similar to yeast data, due to the large size of the calculated correlation set, by executing our binning strategy, a subset of this computed set was employed for our experiments and for the evaluation of gene FS measures. This process resulted in a co-expression dataset of 62 470 gene-gene pairs.

### 3.2 Pretraining of GO-term embeddings

**Fig. 3.**
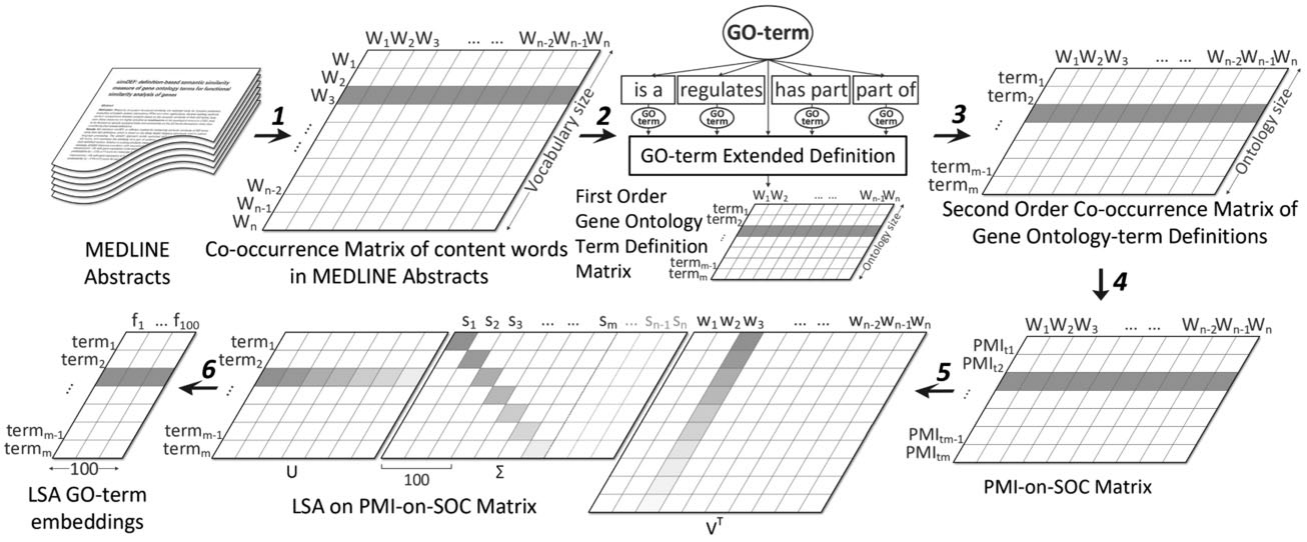
Definition-based embedding model of the Gene Ontology terms

Initialization of a neural network with pretrained embeddings has proven to be effective in a variety of applications (Xu *et al.*, 2015). Our GO term pretraining approach consists of six steps depicted in Figure 3. In essence, the second-order computation of vector representation of GO terms based on their text definitions and co-occurrence vector of their content words in MEDLINE abstracts prevents the issue of the sparsity of word features in the first-order vector representation of the definitions (Pesaranghader *et al.*, 2013a); Pointwise Mutual Information statistically defines the degree of association between each GO term and its second-order word features, and Latent Semantic Analysis condenses the final high-dimensional vectors to a size tractable by deep neural networks. Theses pretraining steps are thoroughly detailed in Supplementary material S1.

### 3.3 deepSimDEF network definition

**Fig. 4.**
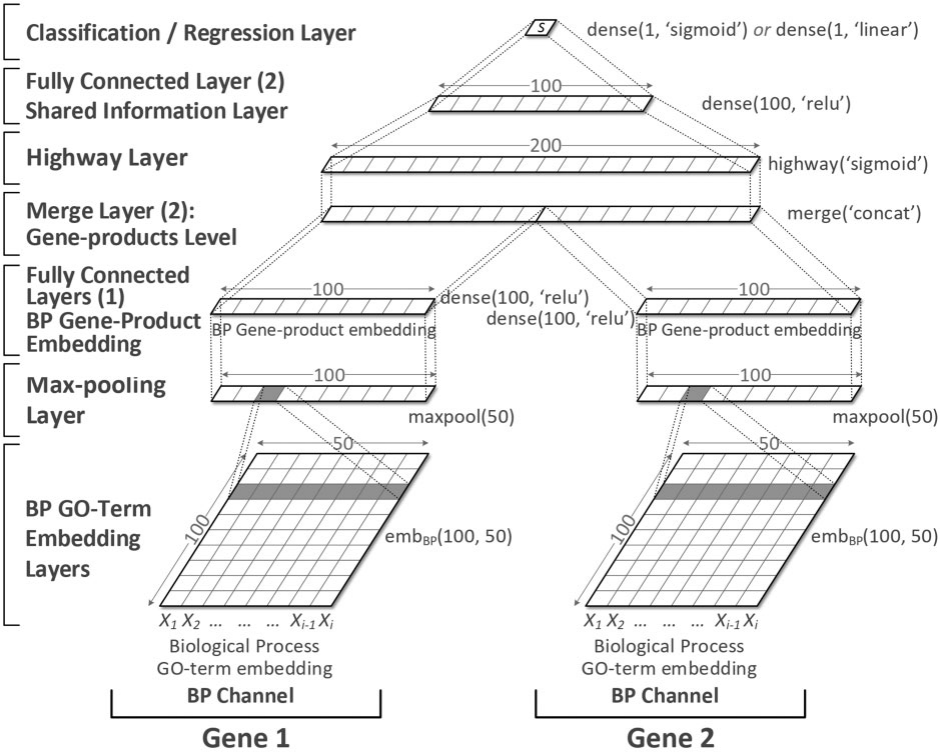
Paired single-channel deepSimDEF network architecture for BP

**Fig. 5.**
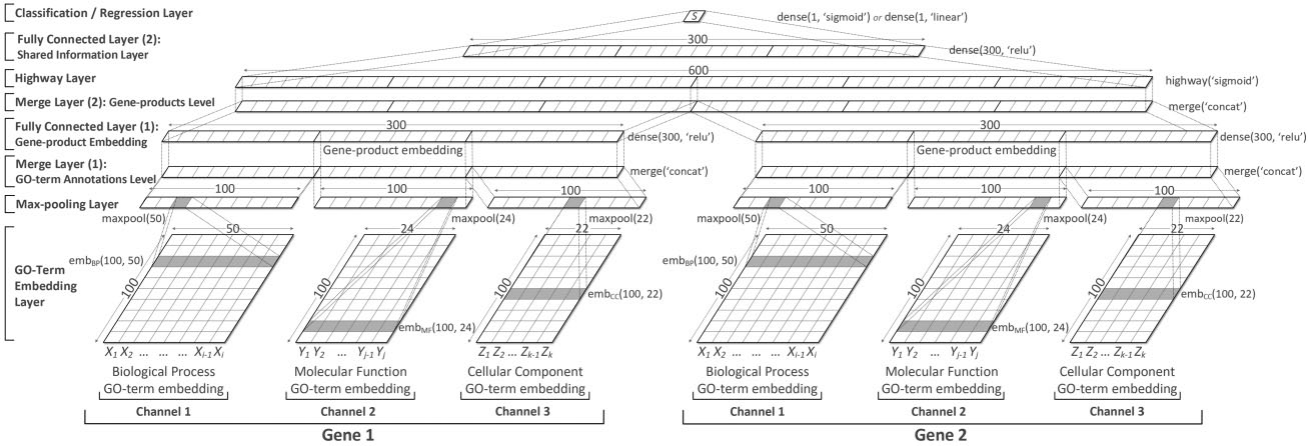
Paired multi-channel deepSimDEF network architecture

deepSimDEF offers *single-channel* and *multi-channel* network architectures that learn and represent the shared information of two proteins based on their GO annotations, and then measure FS of genes for an application of interest. While a single-channel network only considers annotations of one sub-ontology, as depicted in Figure 4 for the BP sub-ontology, the multi-channel architecture, with more layers shown in Figure 5, takes into account all the three GO sub-ontologies together. The 8 layers fundamental to both deepSimDEF architectures are described as follows.

***GO-term* *embedding* *layer.*** The GO term annotations of two proteins are fed to the model as indexes taken from three fixed sets of GO_BP,_ GO_CC_ or/and GO_MF._ These sets contain the indexed GO terms of a particular database from the sub-ontologies of BP, CC and MF. Each set is also associated with a 100-row *look-up table*. These tables, ideally initialized with our pretrained GO-term embeddings, are parameters of the model. First, for every protein, its GO term indexes transform into vectors by looking up their GO-term embeddings. Then, within the embedding layer, for each sub-ontology, the two input proteins are represented as two lists of fixed length *t*_0_, each list containing the 100-dimensional GO embeddings of those two genes’ annotations looked up already (Eq. (3)). In the architectures, for consistency across GO annotations of all genes, whenever the annotation sets of a gene had the length of less than *t*_0_, we padded the annotation list with a generic vector of a large negative value (padding was repeated whenever needed); subsequent Max-pooling Layer later suppressed the effect of this generic vector, and the final estimations were calculated only based on the actual annotations. For yeast database, when IEA–, inferred from the largest number of annotation for a gene, the fixed annotation length for BP, CC and MF were 50, 22 and 24, respectively.

(3) $$X_{\mathrm{ebm}}=[x_{1},x_{2},\ldots ,x_{t_{0}}]\in R^{100\times t_{0}}$$

where $x_{i}$ denotes the GO-term embedding of the *i*th BP GO annotation of a gene. An embedding layer is denoted by *ebm(100, t_0_)* in the figures.

***Max-pooling* *layer.*** Generally, a max-pooling layer aggregates the input vectors by taking the maximum over a set of intervals. Here, for the output of an embedding layer, the max operation is applied over all column features, which is denoted by *maxpool(t_0_)*. We also considered *flattening* of the resulting pooled column-vector into a row feature-vector representation as an integrated part of the max-pooling layer prior to passing the results of the layer to a higher fully connected layer. After max-pooling, proteins with different lengths of GO annotations are represented with 100-dimensional global feature vectors each for one sub-ontology.

***Merge* *layer.*** For a single-channel architecture, we have only one merge layer at the gene-product similarity level due to the paired nature of the input data. That means prior to the extraction and representation of the shared information between two gene products, their individual feature vectors need to be merged through *concatenation*. For the multi-channel architecture, however, besides having a merge layer at the gene-product level, we have an earlier merge layer at the GO term annotations level. For a given gene product of an input gene pair, this extra merge layer is used to concatenate the three 100-dimensional feature vectors of the BP, CC and MF annotations from the max-pooling layer. In the multi-channel architecture, at the GO term annotation level, $m_{go\_\mathrm{multi}}\in R^{1\times 300}$ is the result of the merge layer. At the gene-product level, $m_{gp\_\mathrm{single}}\in R^{1\times 200}$ and $m_{gp\_\mathrm{multi}}\in R^{1\times 600}$ are the results of the merge layers for the paired single-channel and paired multi-channel architectures, respectively. Merge layers are denoted by *merge(‘concat’)*.

***Fully*** ***connected* *layer.*** The fully connected layer takes a *d*_0_-dimensional input vector $x_{\mathrm{fch}}\in R^{d_{0}}$ to learn higher level feature representations of the underneath layers (In the equations, · denotes matrix multiplication.) with:

(4) $$h=\mathrm{ReLU}(W_{h}\cdot x_{\mathrm{fch}}+b_{h}),$$

where $W_{h}\in R^{n_{\mathrm{hid}}\times d_{0}}$, *n*_hid_ is the size of the fully connected hidden layer, $b_{h}\in R^{n_{\mathrm{hid}}}$ is the bias vector, and ReLU is the rectified linear activation function (Nair and Hinton, 2010). The output of the first fully connected layer can be seen as the embeddings of the input gene products. Depending on whether the single-channel or multi-channel network is employed, this embedding size can be 100-dimensional or 300-dimensional. The fully connected hidden layers are denoted by *dense(n_hid_, ‘relu’)*. At the similarity level, the fully connected layer improves representation of the shared information between two genes.

***Highway* *layer.*** In the previous measures including simDEF, for FS estimation of two input gene products, human-engineered aggregation metrics were used—while the SS scores of their pair-wise GO annotations made the inputs of these metrics. However, there is no consensus in the literature on what metric is the best choice for the aggregation of the shared information, as from one biological experiment to another the results vary, and even sometimes, the conclusions contradict each other (Guzzi *et al.*, 2012). In the deepSimDEF model, the highway layer (Srivastava *et al.*, 2015) is devised to let the model itself properly learn an adaptive representation of the provided information of the two input genes encoded in the lower layer for the comparison of those genes’ biological traits including their molecular functions (see also Supplementary material S1). This representation uses a *gating mechanism* that controls the flow of information from the two gene products into an aggregated high-level representation. This adaptive representation of the shared information strengthens an affine transformation—similar to what is presented in Eq. (4)—with a non-linear transform function ***T***. We refer to the vector ***T*** as the transform gate since it expresses how the output is produced through *transforming* or *carrying* the input. If we consider the size of the concatenated feature vectors of two input genes to be *d*_1_-dimensional, ***T*** can be formulated as:

(5) $$T=\sigma (W_{T}\cdot x_{\mathrm{fch}}+b_{T}),$$

where $W_{T}\in R^{n_{\mathrm{hid}}\times d_{1}}$ is the weight matrix, *n*_hid_ is the size of the fully connected hidden layer and here is equal to *d*_1_ since we do not want to expand or shrink the representation result at this stage, $b_{T}\in R^{n_{\mathrm{hid}}}$ is the bias vector, and *σ* is a *sigmoid function* employed in the original paper as the transform function (Srivastava *et al.*, 2015). If we want to represent two extreme cases which apply either transform state or block (or carry) state on the input data, Eq. (6) formulates that for us:

(6) $$x\prime =\{\begin{array}{cc} x_{\mathrm{fch}}, & if\;T=0\\ \sigma (W_{h}\cdot x_{\mathrm{fch}}+b_{h}), & if\;T=1 \end{array}.$$

Therefore, depending on the output of the transform gates, a highway layer should smoothly vary its behavior between that of a plain layer with a non-linear activation of interest (if ***T ***=*** ***1; in deepSimDEF we achieved better results with sigmoid function) and that of a layer which simply passes its inputs through (if ***T ***=*** ***0). Equation (7) formulates this favorable behavior (In the equation, ⊙ implies element-wise multiplication.):

(7) $$x\prime =\sigma (W_{h}\cdot x_{\mathrm{fch}}+b_{h})⊙T+x_{\mathrm{fch}}⊙(1-T).$$

The transform gate—which is the principal component in the deepSimDEF network(s) for a high-level representation of the shared information of two input genes, and all the weights in the highway layer, will be learned during the training phase. The highway layer is denoted by *highway(‘sigmoid’)*.

***Classification/r* *egression* *layer.*** Whether an experiment conducted in a study is a classification problem or a regression problem, the output of the last dense layer is fully connected to either a *sigmoid classification* layer (e.g. for our PPI experiment) or a *linear regression* layer (for the gene expression and sequence homology experiments). After the lower layer processing, a fixed dimensional feature vector $x_{cl}$ or $x_{rg}\in R^{d_{2}}$ is the input to the classification/regression layer, with a sigmoid or linear activation, whose output is the FS estimation of the genes. For a classification task we have:

(8) $$p(y=i|x_{cl})=\frac{\;\text{exp}(W_{out_{i}}\cdot x_{cl}+b_{out_{i}})}{\sum_{j=1}^{n_{\mathrm{out}}}exp(W_{out_{j}}\cdot x_{cl}+b_{out_{j}})},$$

where $p(y=i|x_{cl})$ outputs probability distribution over labels, $W_{\mathrm{out}}\in R^{n_{\mathrm{out}}\times d_{2}}$, *n*_out_ is the size of the classification layer (for the PPI prediction it is equal to two types), $b_{\mathrm{out}}\in R$ is the bias vector, and *d*_2_ is either 100-dimensional (for single-channel) or 300-dimensional (for multi-channel architecture). The classification layer is denoted by *dense(1, ‘sigmoid’)*. For a regression task:

(9) $$\hat{y}=W_{\mathrm{out}}\cdot x_{rg}+b_{\mathrm{out}},$$

where $\hat{y}$ outputs a scalar value, $W_{\mathrm{out}}\in R^{1\times d_{2}}$, *d*_2_ is either 100- or 300-dimensional depending on the architecture, and $b_{\mathrm{out}}\in R$ is the bias vector. The regression layer is denoted by *dense(1, ‘linear’)*.

Since a deepSimDEF network needs to be symmetric to produce the same result for the two input pairs of [*g*_1_, *g*_2_] and [*g*_2_, *g*_1_], all equivalent layers of the paired networks, including embedding layers, must share the same weights [similar to Siamese network (Siamese network is an artificial neural network that uses the same weights while working in tandem on two different input vectors to compute comparable output vectors.)]. Meaning, for each sub-ontology, we only have one look-up table (initialized randomly or with the pretrained GO-term embeddings). In the training phase and during back-propagation, this table(s) will be updated simultaneously for every gene product in a training gene product pair. We also used dropout (Srivastava *et al.*, 2014) of 0.3 on the fully connected and highway layers to allow a more accurate generalization. The parameters of the networks are optimized to maximize the correlation between the estimated FS of gene products predicted by the models and the target scores in the training datasets. This selection was done in a 10-fold cross-validation manner where validation splits chose the best parameters using an early stopping strategy (Prechelt, 1998). Additionally, since the weight matrices of the highway layer for the concatenated feature-vectors of the paired networks are not symmetric and do not update symmetrically, we not only trained the networks on ([*g*_1_, *g*_2_], *score*) instances, we also trained them on ([*g*_2_, *g*_1_], *score*) instances. See Supplementary material S4 for further details and the exact hyper-parameters of the networks.

## 4 Discussion

In comparison to baseline FS measures, validation of deepSimDEF on yeast and human reference datasets yielded increases in PPI predictability by >4.5% and ∼5%, respectively; a correlation improvement of ∼9% and ∼6% with yeast and human gene co-expression values; and improved correlation with sequence homology by up to 6% for both organisms. Unsurprisingly, we observed significantly better results for the predictions in yeast than in human, especially for the co-expression analysis. This is likely due to the fact that our human data came from whole blood samples (the largest dataset available in GTEx), which is a mix of several cell types regulated in different ways, inevitably creating noise in co-expression networks, whereas the information available from yeast co-expression networks is likely more complete given its unicellular nature.

One important aspect regarding the hyper-parameter setting of the deepSimDEF networks was that for all the experiments, one set of hyper-parameters always helped to get the optimal results for the networks (multi- or single-channel). For example, if we changed the embedding size in one experiment and observed a decline or an improvement in the results, for other experiments, we observed the same trend in the results applying the same changes to their networks. This allowed us to maintain a consistent structure across all experiments, which will be very beneficial as deepSimDEF can be extended to less well-characterized datasets than the yeast and human examples we show here. Regarding computational complexity of the deepSimDEF model, since the networks are trained in advance, prediction of FS for batches of new proteins will be very fast at inference time while this is not true for the pair-wise FS measures. deepSimDEF networks were trained and tested on NVIDIA Quadro RTX 6000 GPUs. Depending on the experiment and deepSimDEF network architecture the training time varied from a few hours to almost a day.

Future work with deepSimDEF can involve extension to other problems where FS and SS measures have been applied, including microRNA function analysis (Peng *et al.*, 2017), co-expression network construction (Wang *et al.*, 2017), drug discovery (Sridhar *et al.*, 2016) and cancer treatment studies (Schaefer and Serrano, 2016). deepSimDEF needs to be tested on species other than yeast and human as well, and, in humans, on different tissues. Indeed, co-expression and PPI patterns will vary according to tissue type, and future work should focus on integrating multiple tissues to derive tissue-specific predictions.

Finally, we emphasize that our result derived from IEA should be taken with a grain of salt as such annotations are typically assigned on the basis of homology or domain content, meaning that if two proteins are similar in the sequence they are likely to obtain similar inferred GO annotations. There may thus be an influence of data circularity in the behavior of the results derived from IEA (Pesquita *et al.*, 2008), and it is possible that we are partly measuring the sensitivity of the electronically inferred GO annotations. However, by excluding IEA annotations in our experiments, we could not identify any significant change in the prediction power of deepSimDEF networks. On the other hand, IEA annotations can produce erroneous results when key functional residues are mutated, when genes are duplicated to acquire additional functions, or when the alignment does not span the whole length of the proteins possibly indicating changes in domain architecture. As a result, the computational assignment of GO terms demands more advanced techniques which go beyond homologous sequences. Such techniques are the topic of many recent studies (Littmann *et al.*, 2021; Seyyedsalehi *et al.*, 2021). While we considered IEA+ and IEA− in our experiments (as commonly done in the previous studies), sensitivity to other evidence codes employed in the GO annotations pipeline can be the subject of further studies. Last but not least, in the context of transfer learning, more studies are needed to be done to discover how the learned information from a biological task for an organism can be transferred to another organism.

## 5 Conclusions

Many important applications in computational molecular biology such as gene clustering, protein function prediction, protein interaction evaluation, and disease gene prioritization require functional similarity of genes. deepSimDEF offers a novel deep neural network-based tool for functional similarity prediction of genes and gene products. It results in valuable low-dimensional embeddings of GO terms and gene products and provides powerful, flexible, easily transferable deep neural architectures applicable to a wide range of problems in genomics and proteomics. When evaluated on the yeast and human databases, deepSimDEF single-channel and multi-channel networks outperformed the well-known functional similarity measures in the tasks of PPI prediction, correlation with gene expression as well as correlation with sequence homology data by gaining large margins of improvement. Also, in contrast to previous measures which are computationally expensive, once a deepSimDEF network is trained, its functional similarity prediction of batches of genes is reasonably fast which could be substantially helpful for real-life applications.

## Funding

This work was supported by the Institute for Data Valorization (IVADO)/Genome Quebec grant [PRF-2017-023 to J.H.]; by NSERC CREATE Grant and the grant from Poland’s National Scientific Center available to S.M.; and by NSERC Discovery Grants available to S.M. J.H is a Fonds de la Recherche du Québec en Santé (FRQS) Junior 1 Scholar. M.S. as well. S.M. and R.G.B. were supported by the Canada Research Chairs program.

*Conflict of Interest*: none declared.

## Supplementary Material

btac304_Supplementary_Data
