# Supplementary material for: deepSimDEF: deep neural embeddings of gene products and gene ontology terms for functional analysis of genes
Source: Bioinformatics. 2022 May 10;38(11):3051–61. doi: 10.1093/bioinformatics/btac304 (PMC9154256; doi:10.1093/bioinformatics/btac304)
Supplement: btac304_Supplementary_Data [file btac304_supplementary_data.zip › deepSimDEF_Supplementary_Material_1.pdf]

deepSimDEF: deep neural embeddings of gene products and Gene Ontology terms for functional analysis of genes

(supplementary file 1)

Ahmad Pesaranghader[1,2,3] ✉ Stan Matwin[5,6,8] Marina Sokolova[6,7] Jean-Christophe Grenier[1,2]
Robert G. Beiko[5] and Julie G. Hussin[1,2] ✉

✉ pesarana@mila.quebec, julie.hussin@umontreal.ca
Full list of author information is available at the end of the article

Background

Use cases of functional similarity prediction between genes

Several biomedical and biological problems can benefit from semantic similarity and functional similarity measures (SS and FS, respectively), among which we selected the most common applications to conduct our experiments. These problems include but are not limited to protein subcellular localization prediction [1, 2, 3], protein function prediction [4, 5, 6], co-expression analysis and co-expression network construction [7, 8], inferring protein-protein interactions and PPI network construction [9, 10, 9], biomarker-discovery, disease-discovery, and cancer treatment applications [11, 12, 13, 14], drug-discovery applications [15, 16, 17], and microRNAs function analysis [12, 16, 14].

Briefly discussed, Yang et al. in their study [18] proposed a new method to measure the functional similarity for miRNAs, called miRGOFS with two notable features: (I) it adopts a new GO SS metric which considers both common ancestors and descendants of GO terms; (II) it computes the similarity between GO sets in an asymmetric manner, and weights each GO term by its statistical significance. The showed miRGOFS-based predictor achieves an F1 of 61.2% on a benchmark dataset of miRNA localization, and AUC values of 87.7 and 81.1% on two benchmark sets of miRNA–disease association, respectively. Schaefer and Serrano in their study [12] demonstrated the importance of environmental impact for understanding why certain cancer genes are only involved in the development of some cancer types but are rarely found mutated in other types of cancer. In their study genes were functionally classified based on GO annotations whereas FS of gene pairs was computed as the mean of the SS of GO terms. They argue that the most cancer-general genes tend to be functionally more similar to each other (measured by the distribution of GO functional similarities of all pairs of genes) than the most cancer-specific genes. In another study by Meng et al. [13], for each cancer type, they constructed a miRNA–miRNA functionally synergistic network based on the functions of miRNA targets and their topological features in that cancer PPI network. To identify functionally synergistic miRNA pairs in a specific type of cancer, the functional similarity of targets was first calculated with the help of Resnik measure. Then, the proximity of targets in that cancer PPI network was measured. Along with some other similarity scores, Kim et al. [15] in their study used GO similarity of drug-related genes to address drug repositioning of herbal compounds. To this end, GO terms SS scores were computed with one of the information content-based measures while their functional similarities were calculated with the best-match average (BMA) metric.

deepSimDEF directly estimates FS of genes based on their GO annotations. The pre-trained as well as the fine-tuned embedding vectors of GO terms can be utilised as feature vectors in downstream biological tasks or could be used for SS measurement between GO terms in terms of their Euclidean distance or cosine similarity score.

GO-based semantic similarity measures compared to deepSimDEF

Most early semantic similarity (SS) measures were developed for linguistic studies in natural language processing. Recently, semantic similarity measurement methods have been applied to and further developed and tailored for biological uses as listed below. Considering GO and gene product annotations as information resources, the semantic similarity measures employing these resources are investigated in detail in this supplementary file as follows:

Resnik Measure. Resnik (1995) [19] uses the concept of "information content" (IC) to define a semantic similarity measure. The IC for a term located in an ontology is based on the probability or *p(t)* of occurrence of that term in a corpus.

$$p(t) = \frac{\text{freq}(t)}{\text{freq (root)}} \tag{1}$$

freq(t) is the frequency of t and all its descendants in the ontology summed together. Generally, IC of a term in an ontology indicates how informative that term is in that ontology. As a rule of thumb, the closer to the root, the less informative that term will be. IC of the term t is given by:

$$IC(t) = -\log(p(t)) \tag{2}$$

The more information two terms share, the higher their similarity. The shared information is captured by the set of common ancestors in the graph. The amount of shared information and thus the similarity between the two terms is quantified by the IC of their least common ancestors (LCA). This leads us to the following formula for similarity measurement of two terms in an ontology:

$$\text{sim}_{Resnik}\left(t_1, t_2\right) = \max\left(IC\left(LCA\left(t_1, t_2\right)\right)\right) \tag{3}$$

Jiang and Conrath Measure. Since the Resnik measure considers only the IC of ancestors and ignores input terms' level of specificity, Jiang and Conrath (1997) [20] deal with this issue by taking the IC of the input term into account:

$$\text{sim}_{Jiang}\left(t_1, t_2\right) = 1 + IC\left(LCA\left(t_1, t_2\right)\right) - \frac{IC\left(t_1\right) + IC\left(t_2\right)}{2} \tag{4}$$

Lin Measure. Since Jiang was originally an unnormalized distance measure, Lin (1998) [21] proposed a new similarity measure to resolve that issue:

$$\mathrm{sim}_{Lin}\left(t_1, t_2\right) = \frac{2 \times IC\left(LCA\left(t_1, t_2\right)\right)}{IC\left(t_1\right) + IC\left(t_2\right)} \tag{5}$$

GraSM Measure. Resnik uses the most informative common ancestor (LCA), but GraSM [22] takes into account the average ICs for all disjoint common ancestors instead of choosing only the maximum IC among all the disjoint common ancestors. GraSM assumes that two common ancestors are disjunctive if there are independent paths from both ancestors to the GO term:

$$\mathrm{sim}_{GrasM}\left(t_1, t_2\right) = \mathrm{avg}\left(IC\left(LCA\left(t_1, t_2\right)\right)\right) \tag{6}$$

AIC Measure. AIC or Aggregated Information Content [23] is the latest variation of IC-based semantic similarity measures which considers the aggregate contribution of the ancestors of a GO term to the semantics of that GO term. In their study, they first propose the semantic weight of GO term t as:

$$SW(t) = \frac{1}{1 + e^{-\left(IC(t)\right)^{-1}}} \tag{7}$$

and then, by considering A_x as the ancestor set of term x to the root (including x itself), the semantic value SV(x) of the GO term x is computed by adding the semantic weights of its ancestors:

$$SV(x) = \sum_{t \in A_x} SW(t) \tag{8}$$

Having the above values, the semantic similarity between GO terms t_1 and t_2 based on their aggregate IC is as follows:

$$\mathrm{sim}_{AIC}\left(t_1, t_2\right) = \frac{\sum_{t \in A_1 \cap A_{t_2}} 2 \times SW(t)}{SV\left(t_1\right) + SV\left(t_2\right)} \tag{9}$$

clusteredGO Measure. Recently, Dutta et al. [24] presented a new approach (which we call clusteredGO in our evaluation) that utilized IC of the GO terms and the topological information in GO graph to do GO term clustering. It is done with the consideration of the average ICs of the

Disjunctive common ancestors (DCAs). DCAs is a technique that considers all the common ancestors that do not subsume any other common ancestor. Estimated degree of membership of every GO term to each of the cluster centers is measured which is based on the shortest path lengths from the GO term to the respective cluster centers. For this purpose cluster centers are computed in advance based on the level of association of their GO terms in the GO graph (with an empirical calculation of a threshold for the selection of candidate centers). And at the end a weight parameter on the basis of the maximum difference in membership of two GO terms with respect to all cluster centers is measured, which gives more weightage to GO term pairs having smaller dissimilarity with respect to membership values and vice-versa. These weights are directly used for the computation of ICs with respect to DSAs method. For detailed computation of GO Terms as cluster centers, GO term membership function, calculation of weights and IC values, we refer an avid reader to the Methodology section of the original paper.

simGIC Measure. simGIC or Graph Information Content similarity [25] is a functional similarity of gene products. It directly employs the IC of GO terms associated with two gene products. For two gene products A and B with annotation sets of T_A and T_B, simGIC is given by:

$$\text{simGIC}(A, B) = \frac{\sum_{t \in T_A \cap T_B} IC(t)}{\sum_{t \in T_A \cup T_B} IC(t)} \tag{10}$$

AicInferSentGO Measure. AicInferSentGO is a recent study by Duong et. al. [26] that adopts a known natural language processing (NLP) model called word2vec [27], which converts every word in a corpus into an N-dimensional vector, to measure the similarity of GO terms. This is because in word2vec, vectors of semantically similar words would be closer to each other in the Euclidean (embedding) space while for dissimilar words it is the otherwise.

More specifically, the task to compare two GO terms reduces to the problem of comparing their definitions that are two sentences. Suppose that GO terms T_A and T_B have sentences Z and V as their definitions, respectively. InferSent is a classification model based on the neural network architecture; its full description is given by Conneau et al. [28]. InferSent's first layer is the word vectors for words in the entire training data set (i.e., words in the collection of all GO term definitions). For GO term similarity use, these word vectors should be specific to biology, so, the word2vec vectors trained on PubMed abstracts were used. For measuring similarity of two GO terms using InferSentGO takes two sentences Z and V as one training sample. They defined two categories *entailment* (related pairs in GO or positive cases) and *neutral* (unrelated pairs or negative cases) as random training datasets, and estimated the probability $\mathbb{P}(T_A \text{ entails } T_B)$. This metric allows to gauge the semantic similarity for T_A and T_B. To measure the semantic similarity for two GO terms, we have the metric:

$$\text{InferSentGO } (T_A, T_B) = \max\{\mathbb{P}(T_A \text{ entails } T_B), \mathbb{P}(T_B \text{ entails } T_A)\} \tag{11}$$

ranging from 0 to 1.

Finally, to give more strength to their model by combining InferSentGO with AIC measures as follows:

$$\text{AicInferSentGO}\ (T_A, T_B) = \frac{\text{AIC}(T_A, T_B) +\ \text{InferSentGO}\ (T_A, T_B)}{2} \tag{12}$$

simDEF Measure. simDEF [29] closely follows gloss vector semantic relatedness measure proposed by Pedersen et al. [30]. Generally, this measure constructs definitions (glosses) of the terms from a predefined thesaurus (GO in our case) and estimates the semantic relatedness of two terms using the cosine of the angle between those terms' gloss-vectors. In their approach, every word in the definition of one term gets replaced by its context vector from the co-occurrence data from the corpus, and then all of these context vectors summed together build that term's definition-vector (gloss-vector). The Gloss Vector measure is highly valuable as it employs both terms' definitions and empirical knowledge implicit in a text corpus. The Gloss Vector comprises five steps which simDEF closely follow:

1 Construction of first order co-occurrence matrix by scanning and counting bigram frequencies (i.e. words that cooccur) in the corpus
2 Removing insignificant words using low and high-frequency cut-off points (done by elimination of very low/high frequent bigrams),
3 Using a taxonomy (or a linked thesaurus), developing an extended definition for a term by adding definitions of the directly linked terms to a target term in the taxonomy to the definition of that term,
4 Constructing a definition matrix (all definition vectors) by employing the thresholded first-order matrix from step 2 (cut-off first-order matrix) and the extended definitions from step 3, and finally
5 Estimation of semantic relatedness for a concept-pair (pair of input terms).

Functional similarity measurement of gene product pairs

Upon the computation of GO terms semantic similarities (SS) by either Resnik, Lin, Jiang and Conrath, GraSM, AIC, clusteredGO, simDEF or AicInferSentGO method discussed above, the estimated SS results would be fed into functional similarity (FS) metric of maximum (MAX) or the best-match average (BMA) represented below. T_A and T_B are the sets of GO terms which annotate gene product A and B, respectively:

$$\text{sim}_{\text{MAX}}(A, B) = \text{MAX}_{t_1 \in T_A, t_2 \in T_B}\ (\text{sim}\ (t_1, t_2)) \tag{13}$$

$$\text{sim}_{\text{BMA}}(A, B) = \frac{1}{2}\left(\frac{1}{n}\sum_{t_1 \in T_A} \text{MAX}_{t_1, t_2 \in T_B}\ (\text{sim}\ (t_1, t_2)) + \frac{1}{m}\sum_{t_2 \in T_B} \text{MAX}_{t_1 \in T_A, t_2}\ (\text{sim}\ (t_1, t_2))\right) \tag{14}$$

Results

Importance of the Highway Layer

Prior to working with 'unseen' genes, a deepSimDEF network itself learns how the shared information of two 'known' genes should be transferred to a higher-level representation. This learned representation will ideally dictate the degree of FS association for all genes with respect to a particular biological task or data for which an assumption of gene FS is made already (e.g., the indication of function similarity/connectivity of genes that are closely linked to each other in a PPI network, or the association of homologous genes and their functionality). Even though this aggregation of the shared information can be learned by a fully connected layer, we hypothesized a Highway network (described in Section Methods, Subsection **??**) could do this task more effectively thanks to its gating mechanism. The experiments on the validation and test-split genes supported this notion. As we demonstrate in Table 1, regarding the PPI experiments (yeast and human), we achieved >1% improvement when a Highway network was incorporated into the deepSimDEF architecture; we experienced the same range of improvement when we worked with the gene-expression and sequence homology data.

Table 1 F1-scores for deepSimDEF with a highway network compared to the deepSimDEF with a fully-connected layer in the task of PPI prediction (IEA+)

	deepSimDEF	Including IEA (%)				Excluding IEA (%)			
	Aggregation Layer	ALL	BP	CC	MF	ALL	BP	CC	MF
**Human**	Fully connected	92.01 ± 0.28	89.16 ± 0.27	87.71 ± 0.18	86.15 ± 0.17	91.48 ± 0.31	88.72 ± 0.27	86.42 ± 0.23	85.86 ± 0.18
	Highway network	93.68 ± 0.24	90.62 ± 0.17	89.12 ± 0.26	87.79 ± 0.15	93.12 ± 0.28	90.19 ± 0.29	88.26 ± 0.28	87.38 ± 0.21
**Yeast**	Fully connected	91.18 ± 0.26	90.16 ± 0.21	88.11 ± 0.19	86.03 ± 0.18	91.33 ± 0.24	89.86 ± 0.21	87.47 ± 0.28	85.21 ± 0.26
	Highway network	92.78 ± 0.22	91.57 ± 0.23	89.58 ± 0.27	87.64 ± 0.25	92.99 ± 0.25	91.68 ± 0.25	89.35 ± 0.29	86.69 ± 0.17

Negative control experiments

To evaluate the effect of incorrect annotations of gene products on deepSimDEF model training, we conducted a multi-stage negative control experiment by randomly assigning GO term annotations to the genes in each stage. As presented in Figure 1, first, we fully stripped genes off their original GO term annotations and assigned fully random annotations to them, and conducted experiments listed above (i.e. prediction of PPI, correlation with sequence homology data, and correlation with gene co-expression values), and then, gradually injected correct annotations to the data to observe how the results changed by having more correct GO term annotations assigned. The results improved as we added more correct annotations to the training/test gene data. For example, the results of PPI experiments were ~50% for the F1-score when fully random annotations were given to the genes; once we started to inject correct annotations to the data, they helped with better prediction of interactions. This observation was true for the correlation results of the gene expression and sequence homology experiments as well.

Evaluation of gene product embeddings

To examine the utility and biological relevance of the encoded information of gene products in the gene produce embedding layer of a deepSimDEF network, we conducted a gene classification experiment based on their association with medical subject heading (MeSH) disease categories. For

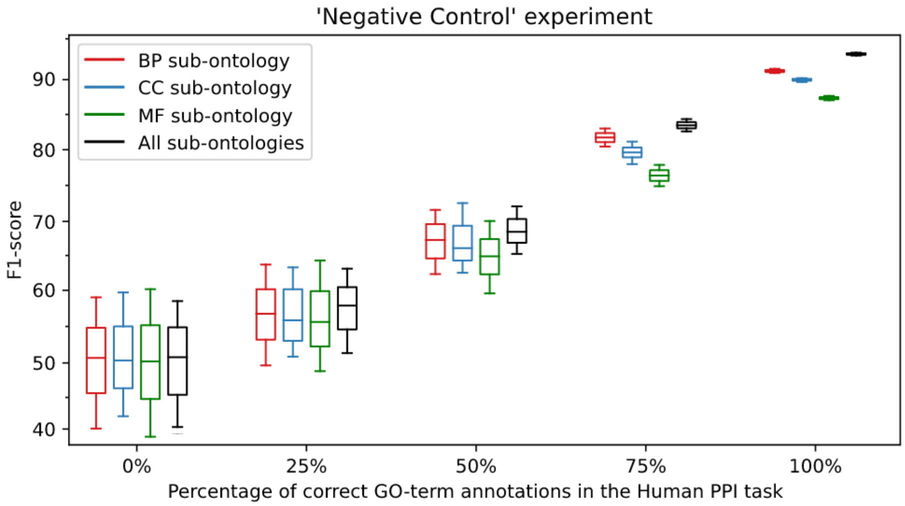

Figure 1 Negative control experiment to verify the importance of correct GO term annotations for a reliable model training (IEA+).
In the experiment we first stripped the examined gene from any correct GO term annotations and assigned completely random GO term annotations to them and then trained and tested the model based on the those GO term annotations. Then, we gradually removed random annotations and replaced them with the original and the correct GO term annotations to see the effect of correct annotations for model training and the prediction of PPIs. Having the embedding layers of the networks initialised with pretrained GO-term embeddings and the rest of weights randomly assigned, we repeated this experiment 10 times to find the mean and the variance of the F1-scores in each consideration.

this purpose, we prepared a list of human genes and their associations with MeSH diseases (and their MeSH disease categories) from DisGeNET platform[1] [31]. Since one gene can be associated with multiple diseases, as well as multiple disease categories or classes, we limited the experiment to those genes which were associated with one disease category in the curated version of the DisGeNET dataset. We observed, compared to random vectors assignment to genes to predict their disease classes with the help of a multi-layer perception classifier, the gene product embeddings from deepSimDEF improved the macro-accuracy and micro- accuracy by up to 3% and 1.5%, respectively.

Methods

Pretraining of GO-term embeddings

Initialisation of a neural network with pretrained embeddings has proven to be effective in a variety of applications [32, 33]. Inspired by studies for (high-dimensional) distributed representation of biomedical concepts [34, 35] and the low-dimensional vector representation of words [36, 37] we pretrained GO-term embeddings in six steps depicted in Figure 2. The pretraining of GO-term embeddings closely followed our approach in the previous work in which we pretrained sense embeddings for every concept in the Unified Medical Language System (UMLS) to address the word sense disambiguation (WSD) of biomedical text data [38]. For pretraining of GO-term embeddings, however, we dealt with GO sub-ontologies, in which GO terms had biologically concerned text definitions that we

[1]https://www.disgenet.org/

represented as low-dimensional vector embeddings. As discussed in Results, these pretrained vectors facilitate and accelerate the exploration and exploitation of training data equipping networks with more accurate predictive power regarding a biological task in hand.

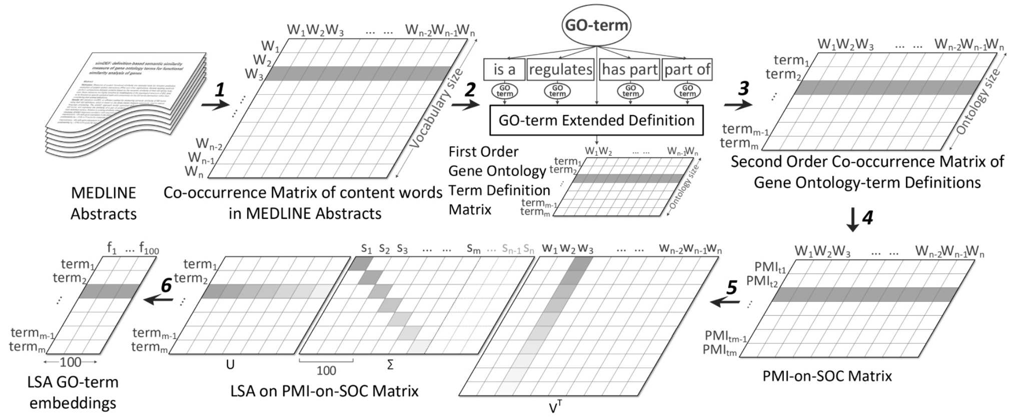

Figure 2 Definition-based embedding model of the Gene Ontology terms.
The pretraining of GO-term embeddings consists of 6 steps. Briefly, the second-order vector representation of GO terms prevents sparsity of word features in the first-order representation of their text definitions; Pointwise Mutual Information statistically defines the degree of association between GO terms and the second-order word features; and Latent Semantic Analysis reduces the result high-dimensional vectors to a size proper for initialisation of a deepSimDEF network.

Step 1 - MEDLINE Word Co-occurrence Matrix. After discarding punctuation, changing all characters to lowercase, and removing stop-words from the MEDLINE bigram list, a list of bigrams and their frequencies for all the content words in the GO term definitions was constructed. We built a co-occurrence matrix from this bigram list of MEDLINE abstracts; a symmetric and sparse matrix that stored contextual information of the MEDLINE words.

Step 2 - Definition Extension and Definition Matrix. In this step, following the simDEF guideline [29], we constructed an extended definition for every GO term. The definition extension of a GO term by the definitions of its neighbor GO term in GO taxonomy, such as their parents and children, enriches that GO term's semantics and to some extent avoids sparsity of the first-order word features in its original and typically brief definition. For this reason, we extended the original definition of every GO term by adding the definitions of the other GO terms which were directly related to that term in the GO structure. The Definition Matrix stored the frequency of the words in every GO-term extended definition. If one word (i.e., W_i word feature) did not appear in a GO term definition the frequency was 0 – which, due to the large size of the word feature space, still could indicate sparsity in these vectors despite the extension. The next step deals with this sparsity with the help of MEDLINE abstract context vector of the content words in the extended definition.

Step 3 - Normalized Second-order Co-occurrence (SOC) Matrix. Each of the W_i word features from the previous step has an associated co-occurrence vector that we computed in Step 1. Following [34, 29, 38], these rich co-occurrence vectors helped to resolve the issue of the sparsity of the first-order definition vectors of the GO terms further through the construction of the second-order vector

presentations of the definitions.
To build a normalized SOC vector of a GO term, we first summed the MEDLINE co-occurrence vectors of the content words in that GO term's extended definition, and then divided the resulting vector by the number of words in that definition (these frequency statistics were stored in the Definition Matrix built in the previous step). In other words, we took the centroid of the co-occurrence vectors associated with the words in one definition, and then normalized the result by the number of constituent vectors in the summation in order to deal with variable lengths of the GO term definitions.

Step 4 - Pointwise Mutual Information (PMI) on SOC Matrix. Not all word features associated with a GO term are equally important [39]. PMI, as in Eq. (15), statistically measures the level of association between one GO term and the word features W_j. This statistical approach is a replacement for the naive consideration of word feature frequency cut-off threshold for the removal of low-frequency occurrences [34]. As a principal rule in NLP, the total frequency of one occurrence indicates how informative that occurrence is, stating the less frequent the occurrence is in a series of events, the more informative that occurrence will be in general [40] – an important consideration ignored in the low-frequency cut-off threshold [39]. PMI on the other hand took these total frequencies into consideration through $p(term_i)$ and $p(W_j)$ probabilities denoted in Eq. (15). Once PMI values were calculated for all the GO terms and word features, we ignored all negative values by changing them to 0 as positive PMI values imply a high semantic correlation of words in a corpus, while a negative PMI value indicates little or no semantic correlation in the corpus [41]. As a common practice in the computation of PMI values, we also applied the Laplace (add-one) smoothing technique to the Normalized SOC Matrix in advance to avoid bias towards infrequent occurrences [42].

$$PMI(term_i, W_j) = log \frac{p(term_i, W_j)}{p(term_i) \times p(W_j)} \tag{15}$$

Step 5 - Latent Semantic Analysis (LSA) on PMI-on-SOC Matrix. LSA is a statistical approach of acquisition and representation of semantics that allows similarities among the elements of a language – such as words or sentences – to be computed based on their co-occurrence patterns in a large corpus [43]; a computational model of meaning that closely mimics human understanding of the contextual use of language widely used for information retrieval and machine understanding of text [44]. Hence, unlike standard keyword-based methods, LSA can detect subtle aspects of semantic content. Employing this statistical approach, formulated by Eq. (16), LSA used *Singular Value Decomposition* (SVD) algorithm that resulted in two square and unitary matrices $\boldsymbol{U}$ an $\boldsymbol{V^T}$, and a non-negative diagonal matrix $\boldsymbol{\Sigma}$ that held singular values on its diagonal in a non-increasing order [45].

$$PMI\_on\_SOC = \boldsymbol{U \Sigma V^T} \tag{16}$$

Step 6 - Reducing the rank of singular values. The reduced dimension semantic representation from LSA allows comparison by computing the semantic similarity between individual terms or groups of terms in a more efficient manner. We use this dimensionality reduction technique to prepare

our well-sized GO-term embeddings for an effective deepSimDEF network initialisation. Having Eq. (17), we truncated the SVD to 100 for low-dimensional representation of GO terms. The resulting matrix (its columns) contained 100 *principal components* of the original matrix. Basically, these principal components are calculated from a covariance matrix which is encoded in $\mathbf{\Sigma}$ in the form of the square root of its eigenvalues (i.e., singular values) [45]. That is, principal components with larger associated variances represent interesting structures, while those with lower variances indicate noise. Determined by our validation sets in the conducted experiments, embedding sizes smaller than 100 yielded worse results whereas higher dimensions did not improve the accuracy and just increased the training time of the networks.

$$GO\_terms\_LSA\_embeddings = \boldsymbol{U}\mathbf{\Sigma}_{100} \tag{17}$$

Author details
[1]Montreal Heart Institute, Montreal, Canada H1T 1C8. [2]Faculty of Medicine, University of Montreal, Montreal, Canada H3T 1J4. [3]Mila - Quebec Artificial Intelligence Institute, Montreal, Canada H2S 3H1. [4]Department of Computer Science and Operations Research, University of Montreal, Montreal, Canada H3T 1J4. [5]Faculty of Computer Science, Dalhousie University, Halifax, Canada B3H 4R2. [6]Institute for Big Data Analytics, Dalhousie University, B3H 4R2 Halifax, Canada. [7]Faculty of Medicine and Faculty of Engineering, University of Ottawa, Ottawa, Canada K1H 8M5. [8]Institute of Computer Science, Polish Academy of Sciences, Warsaw, Poland.

References

1. Cao, Z., Pan, X., Yang, Y., Huang, Y., Shen, H.-B.: The lnclocator: a subcellular localization predictor for long non-coding rnas based on a stacked ensemble classifier. Bioinformatics **1**, 10 (2018)
2. Wan, S., Mak, M.-W., Kung, S.-Y.: Hybridgo-loc: mining hybrid features on gene ontology for predicting subcellular localization of multi-location proteins. PLoS One **9**(3), 89545 (2014)
3. Yang, Y., Lu, B.-L.: Protein subcellular multi-localization prediction using a min-max modular support vector machine. International Journal of Neural Systems **20**(01), 13–28 (2010)
4. Yu, G., Luo, W., Fu, G., Wang, J.: Interspecies gene function prediction using semantic similarity. BMC systems biology **10**(4), 121 (2016)
5. Koskinen, P., Törönen, P., Nokso-Koivisto, J., Holm, L.: Pannzer: high-throughput functional annotation of uncharacterized proteins in an error-prone environment. Bioinformatics **31**(10), 1544–1552 (2015)
6. Yu, G., Zhu, H., Domeniconi, C., Liu, J.: Predicting protein function via downward random walks on a gene ontology. BMC bioinformatics **16**(1), 271 (2015)
7. Makrodimitris, S., Reinders, M.J., Van Ham, R.C.: Metric learning on expression data for gene function prediction. Bioinformatics **36**(4), 1182–1190 (2020)
8. Wang, J., Ma, Z., Carr, S.A., Mertins, P., Zhang, H., Zhang, Z., Chan, D.W., Ellis, M.J., Townsend, R.R., Smith, R.D., *et al.*: Proteome profiling outperforms transcriptome profiling for coexpression based gene function prediction. Molecular & Cellular Proteomics **16**(1), 121–134 (2017)
9. Chapple, C.E., Robisson, B., Spinelli, L., Guien, C., Becker, E., Brun, C.: Extreme multifunctional proteins identified from a human protein interaction network. Nature communications **6**, 7412 (2015)
10. Yu, B., Lou, L., Li, S., Zhang, Y., Qiu, W., Wu, X., Wang, M., Tian, B.: Prediction of protein structural class for low-similarity sequences using chou's pseudo amino acid composition and wavelet denoising. Journal of Molecular Graphics and Modelling **76**, 260–273 (2017)
11. Le, D.-H., Dang, V.-T.: Ontology-based disease similarity network for disease gene prediction. Vietnam Journal of Computer Science **3**(3), 197–205 (2016)
12. Schaefer, M.H., Serrano, L.: Cell type-specific properties and environment shape tissue specificity of cancer genes. Scientific reports **6**, 20707 (2016)
13. Meng, X., Wang, J., Yuan, C., Li, X., Zhou, Y., Hofestädt, R., Chen, M.: Cancernet: a database for decoding multilevel molecular interactions across diverse cancer types. Oncogenesis **4**(12), 177–177 (2015)
14. Biswas, P., Mukhopadhyay, A.: Identifying cancer-associated modules from microrna co-expression networks: a multiobjective evolutionary approach. Soft Computing **24**, 17365–17376 (2020)
15. Kim, E., Choi, A.-s., Nam, H.: Drug repositioning of herbal compounds via a machine-learning approach. BMC bioinformatics **20**(10), 33–43 (2019)
16. Wang, J., Meng, F., Dai, E., Yang, F., Wang, S., Chen, X., Yang, L., Wang, Y., Jiang, W.: Identification of associations between small molecule drugs and mirnas based on functional similarity. Oncotarget **7**(25), 38658 (2016)
17. Li, P., Huang, C., Fu, Y., Wang, J., Wu, Z., Ru, J., Zheng, C., Guo, Z., Chen, X., Zhou, W., *et al.*: Large-scale exploration and analysis of drug combinations. Bioinformatics **31**(12), 2007–2016 (2015)
18. Yang, Y., Fu, X., Qu, W., Xiao, Y., Shen, H.-B.: Mirgofs: a go-based functional similarity measurement for mirnas, with applications to the prediction of mirna subcellular localization and mirna–disease association. Bioinformatics **34**(20), 3547–3556 (2018)
19. Resnik, P.: Using information content to evaluate semantic similarity in a taxonomy. arXiv preprint cmp-lg/9511007 (1995)

20. Jiang, J.J., Conrath, D.W.: Semantic similarity based on corpus statistics and lexical taxonomy. arXiv preprint cmp-lg/9709008 (1997)
21. Lin, D.: An information-theoretic definition of similarity. In: Icml, vol. 98, pp. 296–304 (1998). Citeseer
22. Couto, F.M., Silva, M.J.: Disjunctive shared information between ontology concepts: application to gene ontology. Journal of biomedical semantics **2**(1), 5 (2011)
23. Song, X., Li, L., Srimani, P.K., Philip, S.Y., Wang, J.Z.: Measure the semantic similarity of go terms using aggregate information content. IEEE/ACM transactions on computational biology and bioinformatics **11**(3), 468–476 (2014)
24. Dutta, P., Basu, S., Kundu, M.: Assessment of semantic similarity between proteins using information content and topological properties of the gene ontology graph. IEEE/ACM transactions on computational biology and bioinformatics **15**(3), 839–849 (2018)
25. Pesquita, C., Faria, D., Bastos, H., Falcao, A., Couto, F.: Evaluating go-based semantic similarity measures. In: Proc. 10th Annual Bio-Ontologies Meeting, vol. 37, p. 38 (2007)
26. Duong, D., Ahmad, W.U., Eskin, E., Chang, K.-W., Li, J.J.: Word and sentence embedding tools to measure semantic similarity of gene ontology terms by their definitions. Journal of Computational Biology (2018)
27. Mikolov, T., Sutskever, I., Chen, K., Corrado, G.S., Dean, J.: Distributed representations of words and phrases and their compositionality. In: Advances in Neural Information Processing Systems, pp. 3111–3119 (2013)
28. Conneau, A., Kiela, D., Schwenk, H., Barrault, L., Bordes, A.: Supervised learning of universal sentence representations from natural language inference data. arXiv preprint arXiv:1705.02364 (2017)
29. Pesaranghader, A., Matwin, S., Sokolova, M., Beiko, R.G.: simdef: definition-based semantic similarity measure of gene ontology terms for functional similarity analysis of genes. Bioinformatics **32**(9), 1380–1387 (2016)
30. Pedersen, T., Patwardhan, S., Michelizzi, J.: Wordnet:: Similarity: measuring the relatedness of concepts. In: Demonstration Papers at HLT-NAACL 2004, pp. 38–41 (2004). Association for Computational Linguistics
31. Piñero, J., Ramírez-Anguita, J.M., Saüch-Pitarch, J., Ronzano, F., Centeno, E., Sanz, F., Furlong, L.I.: The disgenet knowledge platform for disease genomics: 2019 update. Nucleic acids research **48**(D1), 845–855 (2020)
32. Chen, D., Manning, C.: A fast and accurate dependency parser using neural networks. In: Proceedings of the 2014 Conference on Empirical Methods in Natural Language Processing (EMNLP), pp. 740–750 (2014)
33. Xu, K., Ba, J., Kiros, R., Cho, K., Courville, A., Salakhudinov, R., Zemel, R., Bengio, Y.: Show, attend and tell: Neural image caption generation with visual attention. In: International Conference on Machine Learning, pp. 2048–2057 (2015)
34. Liu, Y., McInnes, B.T., Pedersen, T., Melton-Meaux, G., Pakhomov, S.: Semantic relatedness study using second order co-occurrence vectors computed from biomedical corpora, umls and wordnet. In: Proceedings of the 2nd ACM SIGHIT International Health Informatics Symposium, pp. 363–372 (2012). ACM
35. Pesaranghader, A., Pesaranghader, A., Rezaei, A., Davoodi, D.: Gene functional similarity analysis by definition-based semantic similarity measurement of go terms. In: Canadian Conference on Artificial Intelligence, pp. 203–214 (2014). Springer
36. Levy, O., Goldberg, Y.: Neural word embedding as implicit matrix factorization. In: Advances in Neural Information Processing Systems, pp. 2177–2185 (2014)
37. Baroni, M., Dinu, G., Kruszewski, G.: Don't count, predict! a systematic comparison of context-counting vs. context-predicting semantic vectors. In: Proceedings of the 52nd Annual Meeting of the Association for Computational Linguistics (Volume 1: Long Papers), vol. 1, pp. 238–247 (2014)
38. Pesaranghader, A., Matwin, S., Sokolova, M., Pesaranghader, A.: deepbiowsd: effective deep neural word sense disambiguation of biomedical text data. Journal of the American Medical Informatics Association (2019). doi:10.1093/jamia/ocy189
39. Pesaranghader, A., Muthaiyah, S., Pesaranghader, A.: Improving gloss vector semantic relatedness measure by integrating pointwise mutual information: Optimizing second-order co-occurrence vectors computed from biomedical corpus and umls. In: 2013 International Conference on Informatics and Creative Multimedia, pp. 196–201 (2013). IEEE
40. Rajaraman, A., Ullman, J.D.: Mining of Massive Datasets. Cambridge University Press, ??? (2011)
41. Yao, L., Mao, C., Luo, Y.: Graph convolutional networks for text classification. In: Proceedings of the AAAI Conference on Artificial Intelligence, vol. 33, pp. 7370–7377 (2019)
42. Dave, K., Lawrence, S., Pennock, D.M.: Mining the peanut gallery: Opinion extraction and semantic classification of product reviews. In: Proceedings of the 12th International Conference on World Wide Web, pp. 519–528 (2003). ACM
43. Nicodemus, K.K., Elvevåg, B., Foltz, P.W., Rosenstein, M., Diaz-Asper, C., Weinberger, D.R.: Category fluency, latent semantic analysis and schizophrenia: a candidate gene approach. Cortex **55**, 182–191 (2014)
44. Landauer, T.K., Foltz, P.W., Laham, D.: An introduction to latent semantic analysis. Discourse processes **25**(2-3), 259–284 (1998)
45. Golub, G.H., Reinsch, C.: Singular value decomposition and least squares solutions. Numerische mathematik **14**(5), 403–420 (1970)
