# Supplementary material for: deepSimDEF: deep neural embeddings of gene products and gene ontology terms for functional analysis of genes
Source: Bioinformatics. 2022 May 10;38(11):3051–61. doi: 10.1093/bioinformatics/btac304 (PMC9154256; doi:10.1093/bioinformatics/btac304)
Supplement: btac304_Supplementary_Data [file btac304_supplementary_data.zip › deepSimDEF_Supplementary_Material_2.pdf]

deepSimDEF: deep neural embeddings of gene products and Gene Ontology terms for functional analysis of genes

(supplementary file 2)

Ahmad Pesaranghader[1,2,3] ✉ Stan Matwin[5,6,8] Marina Sokolova[6,7] Jean-Christophe Grenier[1,2] Robert G. Beiko[5] and Julie G. Hussin[1,2] ✉

✉ pesarana@mila.quebec, julie.hussin@umontreal.ca

Full list of author information is available at the end of the article

Semantic Similarity of Pretrained GO-term Embeddings

Sense similarity, adopted by many studies, is an evaluation approach to see how well the pretrained embeddings are semantically [1, 2]. In essence, our pretraining method organizes embeddings of the GO terms within a Euclidean space based on those GO terms' semantics (arranging books in a physical library is an appropriate analogy for this attempt). Once introduced to a network, these embeddings put that network in a proper state prior to training leading to faster convergence and more accurate results. For three randomly selected GO terms from a pool of >4,000 cellular component (CC) terms and from a pool of >12,000 molecular function (MF) terms, Table 1 and 2 show the 5 top-most similar GO terms to those terms drawn from our pretrained GO-term embeddings using *cosine* similarity (in the library analogy they are similar books arranged next to the given book title). We can see for a given GO-term query, the returned GO terms are very close conceptually.

Table 1 Sense similarity results for three CC terms over pretrained embeddings

Query	GO term ID	GO term Name
**Q #1**	**GO:0000109**	**nucleotide-excision repair complex**
1	GO:0033061	DNA recombinase mediator complex
2	GO:0009380	excinuclease repair complex
3	GO:0019812	type I site-specific deoxyribonuclease complex
4	GO:1990391	DNA repair complex
5	GO:1990249	nucleotide-excision repair, DNA damage recognition complex
**Q #2**	**GO:0000306**	**extrinsic component of vacuolar membrane**
1	GO:0032419	extrinsic component of lysosome membrane
2	GO:0019898	extrinsic component of membrane
3	GO:0031312	extrinsic component of organelle membrane
4	GO:0035452	extrinsic component of plastid membrane
5	GO:0031313	extrinsic component of endosome membrane
**Q #3**	**GO:0044611**	**nuclear pore inner ring**
1	GO:0070762	nuclear pore transmembrane ring
2	GO:0044614	nuclear pore cytoplasmic filaments
3	GO:0031080	nuclear pore outer ring
4	GO:0044612	nuclear pore linkers
5	GO:0044615	nuclear pore nuclear basket

Table 2 Sense similarity results for three MF terms over pretrained embeddings

Query	GO term ID	GO term Name
**Q #1**	**GO:0044653**	**dextrin alpha-glucosidase activity**
1	GO:0044654	starch alpha-glucosidase activity
2	GO:0032450	maltose alpha-glucosidase activity
3	GO:0090600	alpha-1,3-glucosidase activity
4	GO:0004558	alpha-1,4-glucosidase activity
5	GO:0033919	glucan 1,3-alpha-glucosidase activity
**Q #2**	**GO:0071667**	**DNA/RNA hybrid binding**
1	GO:0097098	DNA/RNA hybrid annealing activity
2	GO:0001069	regulatory region RNA binding
3	GO:0003697	single-stranded DNA binding
4	GO:0001067	regulatory region nucleic acid binding
5	GO:1990471	piRNA uni-strand cluster binding
**Q #3**	**GO:0000034**	**adenine deaminase activity**
1	GO:0008892	guanine deaminase activity
2	GO:0004126	cytidine deaminase activity
3	GO:0004131	cytosine deaminase activity
4	GO:0047974	guanosine deaminase activity
5	GO:0035888	isoguanine deaminase activity

Author details
[1]Montreal Heart Institute, Montreal, Canada H1T 1C8. [2]Faculty of Medicine, University of Montreal, Montreal, Canada H3T 1J4. [3]Mila - Quebec Artificial Intelligence Institute, Montreal, Canada H2S 3H1. [4]Department of Computer Science and Operations Research, University of Montreal, Montreal, Canada H3T 1J4. [5]Faculty of Computer Science, Dalhousie University, Halifax, Canada B3H 4R2. [6]Institute for Big Data Analytics, Dalhousie University, B3H 4R2 Halifax, Canada. [7]Faculty of Medicine and Faculty of Engineering, University of Ottawa, Ottawa, Canada K1H 8M5. [8]Institute of Computer Science, Polish Academy of Sciences, Warsaw, Poland.

References

1. Mikolov, T., Sutskever, I., Chen, K., Corrado, G.S., Dean, J.: Distributed representations of words and phrases and their compositionality. In: Advances in Neural Information Processing Systems, pp. 3111–3119 (2013)
2. Pennington, J., Socher, R., Manning, C.D.: Glove: Global vectors for word representation. In: Proceedings of the 2014 Conference on Empirical Methods in Natural Language Processing (EMNLP), pp. 1532–1543 (2014)
