# Supplementary material for: deepSimDEF: deep neural embeddings of gene products and gene ontology terms for functional analysis of genes
Source: Bioinformatics. 2022 May 10;38(11):3051–61. doi: 10.1093/bioinformatics/btac304 (PMC9154256; doi:10.1093/bioinformatics/btac304)
Supplement: btac304_Supplementary_Data [file btac304_supplementary_data.zip › deepSimDEF_Supplementary_Material_3.pdf]

deepSimDEF: deep neural embeddings of gene products and Gene Ontology terms for functional analysis of genes

(supplementary file 3)

Ahmad Pesaranghader[1,2,3] ✉ Stan Matwin[5,6,8] Marina Sokolova[6,7] Jean-Christophe Grenier[1,2] Robert G. Beiko[5] and Julie G. Hussin[1,2] ✉

Baseline functional similarity measure

The baseline FS measures consist of: Resnik [1], Lin [2], Jiang and Conrath [3], GraSM [4], AIC [5], clusteredGO [6], simGIC [7], AicInferSentGO [8], and simDEF [9]. See Additional file 1 for their details.

Author details
[1]Montreal Heart Institute, Montreal, Canada H1T 1C8. [2]Faculty of Medicine, University of Montreal, Montreal, Canada H3T 1J4. [3]Mila - Quebec Artificial Intelligence Institute, Montreal, Canada H2S 3H1. [4]Department of Computer Science and Operations Research, University of Montreal, Montreal, Canada H3T 1J4. [5]Faculty of Computer Science, Dalhousie University, Halifax, Canada B3H 4R2. [6]Institute for Big Data Analytics, Dalhousie University, B3H 4R2 Halifax, Canada. [7]Faculty of Medicine and Faculty of Engineering, University of Ottawa, Ottawa, Canada K1H 8M5. [8]Institute of Computer Science, Polish Academy of Sciences, Warsaw, Poland.

References
1. Resnik, P.: Using information content to evaluate semantic similarity in a taxonomy. arXiv preprint cmp-lg/9511007 (1995)
2. Lin, D.: An information-theoretic definition of similarity. In: Icml, vol. 98, pp. 296–304 (1998). Citeseer
3. Jiang, J.J., Conrath, D.W.: Semantic similarity based on corpus statistics and lexical taxonomy. arXiv preprint cmp-lg/9709008 (1997)
4. Couto, F.M., Silva, M.J.: Disjunctive shared information between ontology concepts: application to gene ontology. Journal of biomedical semantics **2**(1), 5 (2011)
5. Song, X., Li, L., Srimani, P.K., Philip, S.Y., Wang, J.Z.: Measure the semantic similarity of go terms using aggregate information content. IEEE/ACM transactions on computational biology and bioinformatics **11**(3), 468–476 (2014)
6. Dutta, P., Basu, S., Kundu, M.: Assessment of semantic similarity between proteins using information content and topological properties of the gene ontology graph. IEEE/ACM transactions on computational biology and bioinformatics **15**(3), 839–849 (2018)
7. Pesquita, C., Faria, D., Bastos, H., Falcao, A., Couto, F.: Evaluating go-based semantic similarity measures. In: Proc. 10th Annual Bio-Ontologies Meeting, vol. 37, p. 38 (2007)
8. Duong, D., Ahmad, W.U., Eskin, E., Chang, K.-W., Li, J.J.: Word and sentence embedding tools to measure semantic similarity of gene ontology terms by their definitions. Journal of Computational Biology (2018)
9. Pesaranghader, A., Matwin, S., Sokolova, M., Beiko, R.G.: simdef: definition-based semantic similarity measure of gene ontology terms for functional similarity analysis of genes. Bioinformatics **32**(9), 1380–1387 (2016)

Additional Files
Additional file 1
Detailed explanation of the GO-based FS measures compared with deepSimDEF in the study.

Table 1 Pearson's correlation of FS measures with yeast genes co-expressions

	MAX (IEA+)	MAX (IEA-)	BMA (IEA+)	BMA (IEA-)	Jaccard (IEA+)	Jaccard (IEA-)	Highway Layer (IEA+)	Highway Layer (IEA-)
**ALL**								
Resnik	0.5388	0.5163	0.6142	0.5739				
Lin	0.2756	0.3145	0.5819	0.5463				
Jiang and Conrath	0.2502	0.3304	0.581	0.5439				
GraSM	0.5268	0.5183	0.6042	0.5639				
AIC	0.4726	0.4345	0.5919	0.5363				
clusteredGO	0.5145	0.4844	0.5865	0.5415				
simGIC					0.2656	0.2736		
simDEF	0.6076	0.5923	0.6432	0.6259				
AicInferSentGO	0.6098	0.5963	0.6423	0.6235				
deepSimDEF (random emb.)							0.7184	0.7069
deepSimDEF (LSA emb.)							0.7336	0.7227
**BP**								
Resnik	0.5766	0.5623	0.5551	0.5127				
Lin	0.5521	0.5811	0.5542	0.5267				
Jiang and Conrath	0.5682	0.5971	0.5903	0.557				
GraSM	0.5656	0.5663	0.5431	0.5077				
AIC	0.5816	0.5603	0.5501	0.5089				
clusteredGO	0.5736	0.5657	0.5516	0.5347				
simGIC					0.2756	0.2686		
simDEF	0.6036	0.5883	0.6186	0.6023				
AicInferSentGO	0.6068	0.5953	0.6198	0.6053				
deepSimDEF (random emb.)							0.7235	0.7055
deepSimDEF (LSA emb.)							0.7306	0.7218
**CC**								
Resnik	0.5282	0.4801	0.587	0.513				
Lin	0.2842	0.2714	0.4825	0.361				
Jiang and Conrath	0.291	0.2472	0.4417	0.297				
GraSM	0.4882	0.4751	0.5693	0.5023				
AIC	0.5372	0.4841	0.5862	0.5145				
clusteredGO	0.5298	0.4845	0.5879	0.5156				
simGIC					0.262	0.2586		
simDEF	0.5866	0.5783	0.6172	0.5999				
AicInferSentGO	0.5898	0.5853	0.6143	0.5975				
deepSimDEF (random emb.)							0.7183	0.7111
deepSimDEF (LSA emb.)							0.7299	0.7167
**MF**								
Resnik	0.5196	0.5609	0.5139	0.5278				
Lin	0.4081	0.5697	0.4552	0.5501				
Jiang and Conrath	0.2973	0.5854	0.4766	0.5765				
GraSM	0.5216	0.5539	0.5209	0.5538				
AIC	0.5296	0.5619	0.5239	0.5618				
clusteredGO	0.5296	0.5649	0.521	0.5578				
simGIC					0.262	0.2786		
simDEF	0.5796	0.5723	0.6082	0.5919				
AicInferSentGO	0.5828	0.5813	0.6043	0.5985				
deepSimDEF (random emb.)							0.6889	0.6793
deepSimDEF (LSA emb.)							0.7011	0.6939

Table 2 Pearson's correlation of FS measures with human genes co-expressions

	MAX (IEA+)	MAX (IEA-)	BMA (IEA+)	BMA (IEA-)	Jaccard (IEA+)	Jaccard (IEA-)	Highway Layer (IEA+)	Highway Layer (IEA-)
**ALL**								
Resnik	0.1908	0.1916	0.1974	0.1969				
Lin	0.1459	0.1573	0.1815	0.1893				
Jiang and Conrath	0.1186	0.1405	0.1758	0.1861				
GraSM	0.1708	0.1816	0.1944	0.1989				
AIC	0.1489	0.1593	0.1785	0.1853				
clusteredGO	0.1606	0.1625	0.1878	0.1991				
simGIC					0.0901	0.0817		
simDEF	0.2104	0.2236	0.2269	0.2313				
AicInferSentGO	0.2101	0.2208	0.2241	0.2309				
deepSimDEF (random emb.)							0.2741	0.2608
deepSimDEF (LSA emb.)							0.2873	0.2711
**BP**								
Resnik	0.1966	0.1966	0.177	0.1739				
Lin	0.1539	0.1608	0.1588	0.161				
Jiang and Conrath	0.1428	0.1585	0.1711	0.1763				
GraSM	0.1946	0.1946	0.177	0.1839				
AIC	0.1739	0.1768	0.1638	0.179				
clusteredGO	0.1889	0.1888	0.1781	0.1763				
simGIC					0.0601	0.0522		
simDEF	0.1992	0.1961	0.2188	0.2193				
AicInferSentGO	0.1964	0.194	0.2124	0.2149				
deepSimDEF (random emb.)							0.2578	0.2343
deepSimDEF (LSA emb.)							0.2714	0.2741
**CC**								
Resnik	0.1649	0.1329	0.145	0.121				
Lin	0.1248	0.1403	0.1344	0.1295				
Jiang and Conrath	0.1121	0.1368	0.1227	0.1275				
GraSM	0.1629	0.1579	0.155	0.141				
AIC	0.1488	0.1453	0.1394	0.1345				
clusteredGO	0.1609	0.1589	0.1502	0.1482				
simGIC					0.0311	0.0316		
simDEF	0.17012	0.1631	0.1573	0.1388				
AicInferSentGO	0.1651	0.1541	0.1513	0.1355				
deepSimDEF (random emb.)							0.1824	0.1891
deepSimDEF (LSA emb.)							0.2021	0.2088
**MF**								
Resnik	0.1558	0.1727	0.1694	0.1786				
Lin	0.1522	0.1615	0.1442	0.1465				
Jiang and Conrath	0.1421	0.1547	0.1486	0.1466				
GraSM	0.1694	0.1786	0.1558	0.1727				
AIC	0.1522	0.1615	0.1542	0.1565				
clusteredGO	0.1661	0.1727	0.1686	0.1646				
simGIC					0.0512	0.0421		
simDEF	0.1856	0.1899	0.1755	0.1845				
AicInferSentGO	0.1787	0.18810000000000002	0.1655	0.1819				
deepSimDEF (random emb.)							0.1833	0.1391
deepSimDEF (LSA emb.)							0.2132	0.1926

Table 3 Spearman's correlation of FS measures with yeast genes co-expressions

	MAX (IEA+)	MAX (IEA-)	BMA (IEA+)	BMA (IEA-)	Jaccard (IEA+)	Jaccard (IEA-)	Highway Layer (IEA+)	Highway Layer (IEA-)
**ALL**								
Resnik	0.4267	0.367	0.4318	0.3828				
Lin	0.2675	0.2704	0.4232	0.3687				
Jiang and Conrath	0.2457	0.26	0.4263	0.3484				
GraSM	0.4127	0.3625	0.4368	0.3878				
AIC	0.4121	0.3625	0.4348	0.3888				
clusteredGO	0.4105	0.3601	0.4325	0.3808				
simGIC					0.1856	0.1937		
simDEF	0.4611	0.4105	0.4648	0.4312				
AicInferSentGO	0.4602	0.4087	0.459	0.4304				
deepSimDEF (random emb.)							0.527	0.5151
deepSimDEF (LSA emb.)							0.546	0.5313
**BP**								
Resnik	0.424	0.3923	0.4187	0.3875				
Lin	0.4113	0.394	0.4192	0.3941				
Jiang and Conrath	0.3909	0.3737	0.3996	0.376				
GraSM	0.4202	0.3923	0.4087	0.3855				
AIC	0.4212	0.3963	0.4147	0.3847				
clusteredGO	0.4236	0.3903	0.4154	0.3925				
simGIC					0.1942	0.1954		
simDEF	0.4666	0.4353	0.4468	0.4348				
AicInferSentGO	0.4646	0.4322	0.4475	0.4368				
deepSimDEF (random emb.)							0.5391	0.5201
deepSimDEF (LSA emb.)							0.5523	0.5486
**CC**								
Resnik	0.4101	0.3614	0.4271	0.3286				
Lin	0.274	0.2146	0.3889	0.2744				
Jiang and Conrath	0.2614	0.2091	0.3686	0.2461				
GraSM	0.3981	0.3624	0.4221	0.325				
AIC	0.4001	0.3574	0.4221	0.3216				
clusteredGO	0.4121	0.3644	0.4282	0.3255				
simGIC					0.1824	0.1742		
simDEF	0.4521	0.4264	0.4692	0.4085				
AicInferSentGO	0.4541	0.4242	0.4652	0.4055				
deepSimDEF (random emb.)							0.5517	0.5415
deepSimDEF (LSA emb.)							0.559	0.5424
**MF**								
Resnik	0.3925	0.3902	0.3561	0.3699				
Lin	0.343	0.3775	0.3318	0.3621				
Jiang and Conrath	0.332	0.376	0.3485	0.3732				
GraSM	0.3805	0.3872	0.3572	0.3675				
AIC	0.3825	0.3922	0.3542	0.366				
clusteredGO	0.3975	0.3892	0.3526	0.3642				
simGIC					0.1812	0.1902		
simDEF	0.4355	0.4294	0.3954	0.4055				
AicInferSentGO	0.4341	0.4245	0.3967	0.4026				
deepSimDEF (random emb.)							0.5025	0.4973
deepSimDEF (LSA emb.)							0.5183	0.5079

Table 4 Spearman's correlation of FS measures with human genes co-expressions

	MAX (IEA+)	MAX (IEA-)	BMA (IEA+)	BMA (IEA-)	Jaccard (IEA+)	Jaccard (IEA-)	Highway Layer (IEA+)	Highway Layer (IEA-)
**ALL**								
Resnik	0.1318	0.1375	0.1231	0.1315				
Lin	0.1423	0.1503	0.1401	0.1546				
Jiang and Conrath	0.1411	0.1566	0.1404	0.1501				
GraSM	0.1403	0.1543	0.1381	0.1436				
AIC	0.1323	0.1521	0.1422	0.1386				
clusteredGO	0.1463	0.148	0.144	0.1501				
simGIC					0.0724	0.0612		
simDEF	0.1763	0.1785	0.1794	0.1891				
AicInferSentGO	0.1753	0.1795	0.1784	0.1881				
deepSimDEF (random emb.)							0.2458	0.2368
deepSimDEF (LSA emb.)							0.2592	0.2816
**BP**								
Resnik	0.1315	0.1235	0.1107	0.104				
Lin	0.1241	0.1212	0.1065	0.103				
Jiang and Conrath	0.1217	0.1278	0.0974	0.1019				
GraSM	0.1285	0.1205	0.1087	0.1024				
AIC	0.1251	0.1232	0.1045	0.1046				
clusteredGO	0.1227	0.1258	0.1074	0.1019				
simGIC					0.0421	0.0387		
simDEF	0.1607	0.1528	0.1524	0.1489				
AicInferSentGO	0.1599	0.1508	0.1444	0.152				
deepSimDEF (random emb.)							0.2247	0.2023
deepSimDEF (LSA emb.)							0.2363	0.2416
**CC**								
Resnik	0.113	0.1127	0.1079	0.1016				
Lin	0.1219	0.1403	0.1247	0.1245				
Jiang and Conrath	0.1297	0.1514	0.127	0.1312				
GraSM	0.1239	0.1413	0.1222	0.1285				
AIC	0.1289	0.1373	0.1201	0.1205				
clusteredGO	0.1329	0.1463	0.1288	0.1295				
simGIC					0.0291	0.0256		
simDEF	0.1489	0.1603	0.1456	0.1455				
AicInferSentGO	0.1472	0.1591	0.1421	0.1492				
deepSimDEF (random emb.)							0.1694	0.1626
deepSimDEF (LSA emb.)							0.1771	0.1955
**MF**								
Resnik	0.1153	0.1398	0.0994	0.1163				
Lin	0.1471	0.1523	0.1248	0.1323				
Jiang and Conrath	0.1519	0.1532	0.1339	0.1368				
GraSM	0.1489	0.1502	0.135	0.1378				
AIC	0.1539	0.1516	0.1285	0.1309				
clusteredGO	0.1509	0.155	0.1401	0.1325				
simGIC					0.0342	0.0291		
simDEF	0.1539	0.1576	0.1445	0.1359				
AicInferSentGO	0.1559	0.1516	0.1475	0.1379				
deepSimDEF (random emb.)							0.1589	0.1393
deepSimDEF (LSA emb.)							0.1649	0.1499

Table 5 **Pearson's correlation of deepSimDEF and other FS measures for three sub-ontologies against yeast sequence homology (RRBS and LRBS) (IEA+)**

		LRBS				RRBS			
		ALL	BP	CC	MF	ALL	BP	CC	MF
Resnik [1]	MAX	0.6888	0.7223	0.6570	0.4117	0.5668	0.6024	0.5907	0.3340
	BMA	0.5760	0.6102	0.5959	0.3154	0.5947	0.6325	0.6018	0.3124
Lin [2]	MAX	0.2479	0.4801	0.2836	0.4077	0.1507	0.3213	0.1980	0.2519
	BMA	0.5166	0.5447	0.4661	0.3725	0.4763	0.5320	0.4289	0.2866
Jiang and Conrath [3]	MAX	0.2898	0.5112	0.1882	0.3969	0.2241	0.3652	0.1277	0.2875
	BMA	0.5386	0.5989	0.4501	0.3817	0.4867	0.5917	0.3995	0.2898
GraSM [4]	MAX	0.2845	0.5159	0.1892	0.3870	0.2210	0.3655	0.1333	0.2803
	BMA	0.5437	0.5974	0.4465	0.3854	0.4850	0.5862	0.3928	0.2957
AIC [5]	MAX	0.2868	0.5015	0.1809	0.3887	0.2293	0.3748	0.1355	0.2782
	BMA	0.5290	0.5900	0.4450	0.3885	0.4783	0.5880	0.3942	0.2954
clusteredGO [6]	MAX	0.2868	0.5015	0.1809	0.3887	0.2293	0.3748	0.1355	0.2782
	BMA	0.5290	0.5900	0.4450	0.3885	0.4783	0.5880	0.3942	0.2954
simGIC [7]		0.2349	0.4514	0.1224	0.3413	0.1828	0.3333	0.0884	0.2265
simDEF [9]	MAX	0.4964	0.7099	0.3972	0.6008	0.4332	0.5750	0.3290	0.4944
	BMA	0.7294	0.7889	0.6492	0.5751	0.6821	**0.7971**	0.6083	0.4860
AicInferSentGO [8]	MAX	0.4831	0.7135	0.3878	0.6052	0.4200	0.5706	0.3202	0.4928
	BMA	0.7408	**0.8068**	0.6456	0.5749	0.6935	0.7841	0.5896	0.4929
deepSimDEF (random emb.)		0.8692	0.8289	0.8093	0.8534	0.8349	0.7895	0.8128	0.7954
deepSimDEF (LSA emb.)		**0.8853**	0.8541	0.8163	0.8717	**0.8564**	0.8019	0.8243	0.8112

Table 6 **Spearman's correlation of deepSimDEF and other FS measures for three sub-ontologies against yeast sequence homology (RRBS and LRBS) (IEA−)**

		LRBS				RRBS			
		ALL	BP	CC	MF	ALL	BP	CC	MF
Resnik [1]	MAX	0.7169	0.5081	0.5705	0.4441	0.3911	0.3277	0.1951	0.2579
	BMA	0.4737	0.4720	0.5209	0.3768	0.3553	0.3785	0.3113	0.1894
Lin [2]	MAX	0.5387	0.6747	0.4796	0.6881	0.4107	0.5290	0.3859	0.5396
	BMA	0.6485	0.6872	0.4694	0.6154	0.5504	0.5946	0.3982	0.4991
Jiang and Conrath [3]	MAX	0.4360	0.6595	0.4460	0.5898	0.2757	0.5201	0.3549	0.4096
	BMA	0.6866	0.7126	0.5007	0.6170	0.5822	0.6093	0.4220	0.4709
GraSM [4]	MAX	0.4285	0.6676	0.4432	0.5992	0.2755	0.5301	0.3575	0.4028
	BMA	0.6879	0.7157	0.5050	0.6239	0.5757	0.6081	0.4248	0.4759
AIC [5]	MAX	0.4285	0.6676	0.4432	0.5992	0.2755	0.5301	0.3575	0.4028
	BMA	0.6879	0.7157	0.5050	0.6239	0.5757	0.6081	0.4248	0.4759
clusteredGO [6]	MAX	0.4285	0.6676	0.4432	0.5992	0.2755	0.5301	0.3575	0.4028
	BMA	0.6879	0.7157	0.5050	0.6239	0.5757	0.6081	0.4248	0.4759
simGIC [7]		0.3764	0.6241	0.4011	0.5439	0.2235	0.4713	0.2994	0.3576
simDEF [9]	MAX	0.4890	0.7047	0.5003	0.6454	0.3313	0.5766	0.4065	0.4512
	BMA	0.7370	**0.7590**	0.5534	0.6697	0.6379	**0.6541**	0.4628	0.5134
AicInferSentGO [8]	MAX	0.4865	0.7049	0.4946	0.6299	0.3187	0.5775	0.4032	0.4580
	BMA	0.7346	0.7542	0.5449	0.6685	0.6360	**0.6540**	0.4628	0.5291
deepSimDEF (random emb.)		0.7438	0.6518	0.5942	0.7126	0.6923	0.5766	0.5405	0.6685
deepSimDEF (LSA emb.)		**0.7974**	0.7247	0.6418	0.7577	**0.7109**	0.5960	0.5519	0.6798

Table 7 Pearson's correlation of deepSimDEF and other FS measures for three sub-ontologies against yeast sequence homology (RRBS and LRBS) (IEA−)

		LRBS				RRBS			
		**ALL**	**BP**	**CC**	**MF**	**ALL**	**BP**	**CC**	**MF**
Resnik [1]	MAX	0.5922	0.5102	0.5315	0.3225	0.3864	0.3678	0.3024	0.2501
	BMA	0.4637	0.4779	0.5365	0.2392	0.4224	0.4385	0.4152	0.1636
Lin [2]	MAX	0.3766	0.5425	0.3846	0.5219	0.2382	0.3786	0.2781	0.3450
	BMA	0.6261	0.7142	0.4646	0.4513	0.5824	0.6166	0.4694	0.3630
Jiang and Conrath [3]	MAX	0.2974	0.5551	0.3653	0.4518	0.1488	0.3983	0.2733	0.2661
	BMA	0.6656	0.7421	0.4850	0.4345	0.6178	0.6589	0.4539	0.3263
GraSM [4]	MAX	0.3037	0.5454	0.3561	0.4444	0.1440	0.4074	0.2658	0.2575
	BMA	0.6601	0.7390	0.4865	0.4392	0.6124	0.6569	0.4563	0.3359
AIC [5]	MAX	0.3037	0.5454	0.3561	0.4444	0.1440	0.4074	0.2658	0.2575
	BMA	0.6601	0.7390	0.4865	0.4392	0.6124	0.6569	0.4563	0.3359
clusteredGO [6]	MAX	0.3037	0.5454	0.3561	0.4444	0.1440	0.4074	0.2658	0.2575
	BMA	0.6601	0.7390	0.4865	0.4392	0.6124	0.6569	0.4563	0.3359
simGIC [7]		0.2519	0.5005	0.3137	0.3978	0.0972	0.3534	0.2219	0.2036
simDEF [9]	MAX	0.4491	0.7035	0.5060	0.6086	0.2923	0.5415	0.4213	0.4104
	BMA	0.8081	**0.8344**	0.6320	0.5885	0.7722	0.8144	0.6081	0.4748
AicInferSentGO [8]	MAX	0.4525	0.7052	0.5236	0.6009	0.3053	0.5572	0.4195	0.4132
	BMA	0.8253	**0.8344**	0.6328	0.5809	0.7581	**0.8183**	0.5979	0.4721
deepSimDEF (random emb.)		0.8715	0.8160	0.8201	0.8497	0.8404	0.7264	0.8176	0.7929
deepSimDEF (LSA emb.)		**0.8827**	0.8374	0.8352	0.8676	**0.8474**	0.7321	0.8291	0.8089

Table 8 Pearson's correlation of deepSimDEF and other FS measures for three sub-ontologies against human sequence homology (RRBS and LRBS) (IEA+)

		LRBS				RRBS			
		**ALL**	**BP**	**CC**	**MF**	**ALL**	**BP**	**CC**	**MF**
Resnik [1]	MAX	0.5590	0.5328	0.3336	0.5901	0.5444	0.5401	0.4311	0.4979
	BMA	0.5743	0.5393	0.3794	0.5521	0.6532	0.6176	0.5445	0.5409
Lin [2]	MAX	0.2789	0.4944	0.3410	0.3293	0.2374	0.4160	0.3007	0.2698
	BMA	0.5352	0.5454	0.4174	0.3886	0.5737	0.5887	0.4533	0.3885
Jiang and Conrath [3]	MAX	0.2799	0.3738	0.3051	0.3319	0.1652	0.3171	0.2312	0.2326
	BMA	0.5437	0.5569	0.4045	0.3961	0.5904	0.6308	0.4146	0.3946
GraSM [4]	MAX	0.2864	0.3852	0.3059	0.3820	0.1632	0.3383	0.2395	0.3194
	BMA	0.5468	0.4021	0.4139	0.5651	0.5862	0.3926	0.4061	0.6344
AIC [5]	MAX	0.2773	0.3819	0.2954	0.3666	0.1570	0.2334	0.2214	0.3202
	BMA	0.5364	0.3975	0.4113	0.5616	0.5946	0.4043	0.4118	0.6365
clusteredGO [6]	MAX	0.2894	0.3338	0.2956	0.3663	0.1725	0.3318	0.2389	0.3154
	BMA	0.5339	0.3930	0.4081	0.5509	0.5869	0.4010	0.4092	0.6333
simGIC [7]		0.2403	0.2896	0.2502	0.3115	0.1130	0.2852	0.1794	0.2721
simDEF [9]	MAX	0.3529	0.4053	0.3727	0.4371	0.2291	0.2996	0.3046	0.3774
	BMA	0.6202	0.5199	0.4651	0.6277	0.6555	0.5392	0.4871	**0.6828**
AicInferSentGO [8]	MAX	0.3433	0.4083	0.3822	0.4373	0.2309	0.3003	0.2921	0.3946
	BMA	0.6235	0.5123	0.4802	**0.6295**	0.6678	0.5376	0.4761	**0.6828**
deepSimDEF (random emb.)		0.6687	0.5595	0.4645	0.6450	0.7154	0.6590	0.6274	0.7004
deepSimDEF (LSA emb.)		**0.6860**	0.5714	0.4950	0.6720	**0.7335**	0.6670	0.6522	0.7132

Table 9 Spearman's correlation of deepSimDEF and other FS measures for three sub-ontologies against human sequence homology (RRBS and LRBS) (IEA−)

		LRBS				RRBS			
		ALL	BP	CC	MF	ALL	BP	CC	MF
Resnik [1]	MAX	0.4447	0.4367	0.2517	0.5012	0.4537	0.4525	0.2752	0.5147
	BMA	0.4806	0.4385	0.2994	0.4610	0.5212	0.4447	0.3680	0.5259
Lin [2]	MAX	0.2593	0.4655	0.3006	0.2737	0.2326	0.4571	0.2880	0.2295
	BMA	0.4558	0.4587	0.3476	0.3283	0.4784	0.4541	0.4124	0.3700
Jiang and Conrath [3]	MAX	0.2439	0.4487	0.2390	0.3240	0.1659	0.4371	0.2037	0.2236
	BMA	0.4353	0.4924	0.3501	0.3220	0.4786	0.4998	0.4012	0.3400
GraSM [4]	MAX	0.2364	0.3241	0.2381	0.4387	0.1696	0.2278	0.2049	0.4370
	BMA	0.4358	0.3233	0.3560	0.4878	0.4869	0.3460	0.3953	0.5038
AIC [5]	MAX	0.2416	0.3219	0.2463	0.4522	0.1688	0.2146	0.2072	0.4294
	BMA	0.4340	0.3239	0.3442	0.4912	0.4793	0.3326	0.4039	0.5025
clusteredGO [6]	MAX	0.2532	0.3240	0.2431	0.4483	0.1654	0.2184	0.2075	0.4345
	BMA	0.4451	0.3206	0.3501	0.4985	0.4811	0.3427	0.3914	0.5047
simGIC [7]		0.1988	0.2801	0.1859	0.3893	0.1185	0.1694	0.1624	0.3846
simDEF [9]	MAX	0.3180	0.3913	0.3064	0.5283	0.2264	0.2879	0.2797	0.4979
	BMA	0.5128	0.3862	0.4268	**0.5664**	0.5400	0.4077	0.4759	0.5670
AicInferSentGO [8]	MAX	0.3223	0.3890	0.3006	0.5099	0.2308	0.2912	0.2708	0.4973
	BMA	0.5013	0.3881	0.4266	0.5535	0.5575	0.4003	0.4632	**0.5698**
deepSimDEF (random emb.)		0.6423	0.5200	0.4138	0.5970	0.6383	0.5323	0.5160	0.6374
deepSimDEF (LSA emb.)		**0.6590**	0.5378	0.4486	0.6185	**0.6513**	0.5440	0.5194	0.6426

Table 10 Pearson's correlation of deepSimDEF and other FS measures for three sub-ontologies against human sequence homology (RRBS and LRBS) (IEA−)

		LRBS				RRBS			
		ALL	BP	CC	MF	ALL	BP	CC	MF
Resnik [1]	MAX	0.5152	0.4926	0.3326	0.5437	0.5203	0.5196	0.4037	0.4700
	BMA	0.5437	0.5123	0.3589	0.4995	0.6325	0.5961	0.4885	0.5159
Lin [2]	MAX	0.2351	0.4506	0.2972	0.2855	0.1936	0.3722	0.2569	0.2260
	BMA	0.4914	0.5016	0.3736	0.3448	0.5299	0.5449	0.4095	0.3447
Jiang and Conrath [3]	MAX	0.2361	0.3300	0.2613	0.2881	0.1214	0.2733	0.1874	0.1888
	BMA	0.4999	0.5131	0.3607	0.3523	0.5466	0.5870	0.3708	0.3508
GraSM [4]	MAX	0.2440	0.2935	0.2705	0.3238	0.1114	0.1829	0.1830	0.2796
	BMA	0.5057	0.3447	0.3571	0.5088	0.5515	0.3588	0.3639	0.5876
AIC [5]	MAX	0.2420	0.2962	0.2711	0.3315	0.1120	0.1984	0.1949	0.2745
	BMA	0.5060	0.3529	0.3696	0.5207	0.5499	0.3536	0.3791	0.5908
clusteredGO [6]	MAX	0.2377	0.2979	0.2533	0.3393	0.1283	0.1960	0.1940	0.2653
	BMA	0.4933	0.3519	0.3666	0.5089	0.5458	0.3504	0.3687	0.5843
simGIC [7]		0.1857	0.2458	0.2062	0.2816	0.0723	0.1491	0.1420	0.2236
simDEF [9]	MAX	0.3104	0.3585	0.3389	0.4062	0.1914	0.2639	0.2603	0.3441
	BMA	0.5650	0.4134	0.4352	**0.5920**	0.6184	0.4187	0.4316	0.6625
AicInferSentGO [8]	MAX	0.3130	0.3656	0.3252	0.3903	0.1949	0.2662	0.2518	0.3333
	BMA	0.5633	0.4260	0.4255	0.5769	0.6081	0.4164	0.4333	**0.6667**
deepSimDEF (random emb.)		0.6602	0.5569	0.4553	0.6277	0.7178	0.6601	0.6235	0.6956
deepSimDEF (LSA emb.)		**0.6747**	0.5680	0.4846	0.6428	**0.7265**	0.6750	0.6400	0.7097
