# Supplementary material for: deepSimDEF: deep neural embeddings of gene products and gene ontology terms for functional analysis of genes
Source: Bioinformatics. 2022 May 10;38(11):3051–61. doi: 10.1093/bioinformatics/btac304 (PMC9154256; doi:10.1093/bioinformatics/btac304)
Supplement: btac304_Supplementary_Data [file btac304_supplementary_data.zip › deepSimDEF_Supplementary_Material_4.pdf]

deepSimDEF: deep neural embeddings of gene products and Gene Ontology terms for functional analysis of genes

(supplementary file 4)

Ahmad Pesaranghader[1,2,3] ✉ Stan Matwin[5,6,8] Marina Sokolova[6,7] Jean-Christophe Grenier[1,2] Robert G. Beiko[5] and Julie G. Hussin[1,2] ✉

✉ pesarana@mila.quebec, julie.hussin@umontreal.ca

Detailed Architectures of single-channel and multi-channel deepSimDEF network

deepSimDEF is the substantial extension of simDEF model [1] using deep learning representation tools. For this purpose, deepSimDEF offers *single-channel* and *multi-channel* network architectures which learn and represent the shared information of two genes or proteins based on their GO annotations, and then measure FS of genes for an application of interest. While a single-channel network only considers annotations of one sub-ontology, as depicted in Figure 2 for the BP sub-ontology, Figure 3 for the CC sub-ontology, 4 for the MF sub-ontology, the multi-channel architecture, with more layers shown in Figure 1, takes into account all the three GO sub-ontologies together. The several layers fundamental to both deepSimDEF architectures are depicted as follows (final hyper-parameters). The main hyper-parameters of the networks included: embedding size, non-linearity function, hidden layer sizes and numbers, drop-out rate, number of epoch for traning, and learning rate. Additionaly, the code for these architecture is available on GitHub at https://github.com/ahmadpgh/deepSimDEF.

Author details

[1]Montreal Heart Institute, Montreal, Canada H1T 1C8. [2]Faculty of Medicine, University of Montreal, Montreal, Canada H3T 1J4. [3]Mila - Quebec Artificial Intelligence Institute, Montreal, Canada H2S 3H1. [4]Department of Computer Science and Operations Research, University of Montreal, Montreal, Canada H3T 1J4. [5]Faculty of Computer Science, Dalhousie University, Halifax, Canada B3H 4R2. [6]Institute for Big Data Analytics, Dalhousie University, B3H 4R2 Halifax, Canada. [7]Faculty of Medicine and Faculty of Engineering, University of Ottawa, Ottawa, Canada K1H 8M5. [8]Institute of Computer Science, Polish Academy of Sciences, Warsaw, Poland.

References

1. Pesaranghader, A., Matwin, S., Sokolova, M., Beiko, R.G.: simdef: definition-based semantic similarity measure of gene ontology terms for functional similarity analysis of genes. Bioinformatics **32**(9), 1380–1387 (2016)

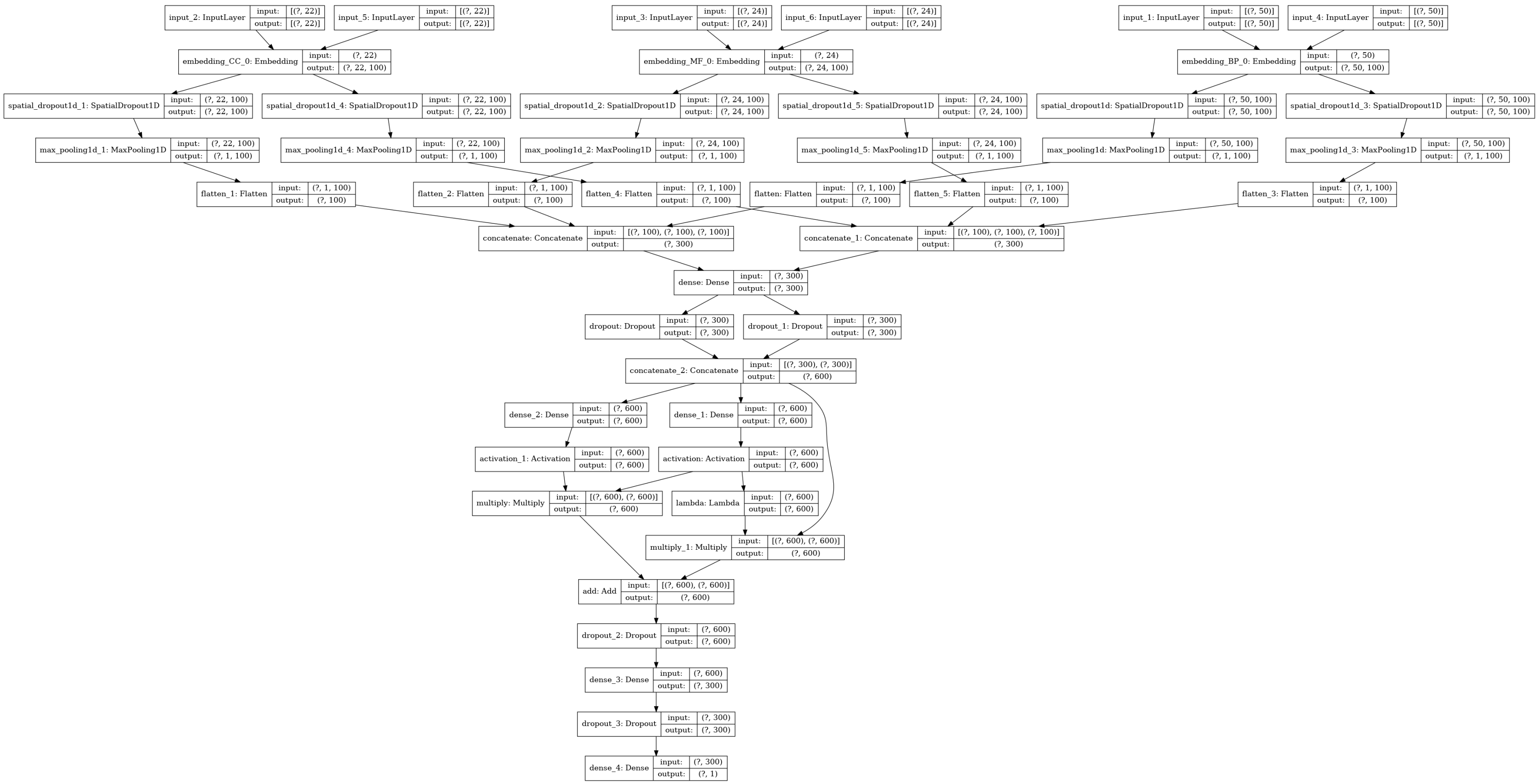

Figure 1 Paired multi-channel deepSimDEF network architecture.
The paired multi-channel deepSimDEF architecture consists of several layers for functional similarity measurement of genes and gene products using their Gene Ontology term annotations from all three GO sub-ontologies. For two input genes, their full GO annotations are fed to the network in the first layer in which they will be represented as six lists of 100-dimensional embedding vectors while every two of them share the same weight. After applying dropout (0.15), max-pooling layer condenses each of these six lists into a 100-dimensional row-vector, and subsequently the first merge layer concatenates them into two rich row-vectors regarding the gene and the sub-ontologies they come from. The second merge layer as well as highway layer together encode the degree of shared information of these two pooled and then merged vectors. In different locations of the architecture, fully-connected layers are considered for better representation of their underlying layers (followed by dropout of 0.3). Finally, based on the biological application in hand, the result of the prediction network is computed which can be either a scalar or a classification probability. The weights of the two pairs are shared during the network training and testing. In an attempt to increase accuracy of the predictions, in contrast to single-channel architecture, this architecture considers all GO annotations from all three sub-ontologies of BP, CC and MF. The figure represented for the yeast species, in the first layer, 50, 22, and 24 are the maximum number of annotations from BP, CC, and MF respectively (any number larger than these would yield the same result from the network due the following max-pooling layer). Depending on the species of choice (e.g. human) these numbers would be different while the rest of the network stays the same.

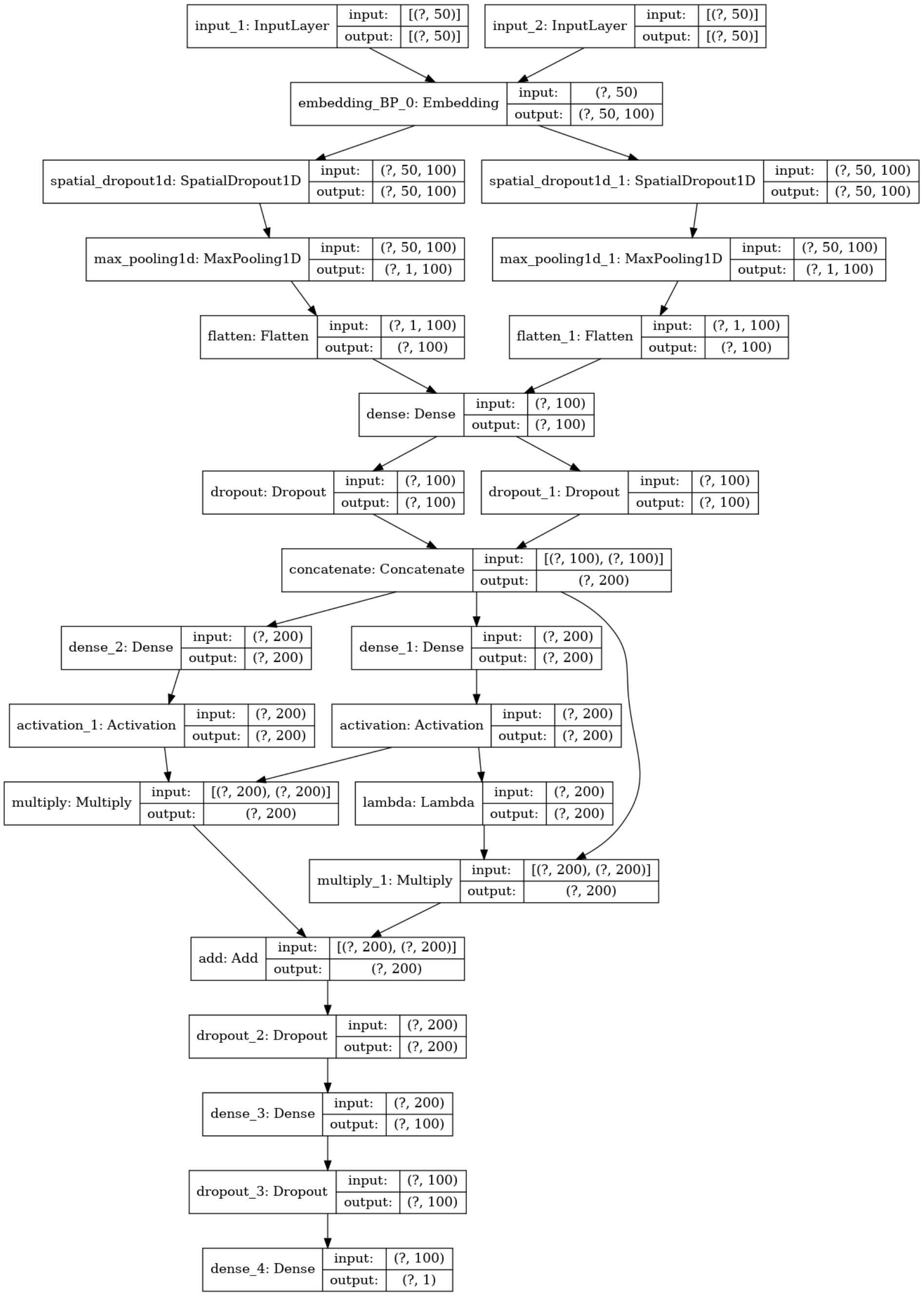

Figure 2 Paired single-channel deepSimDEF network architecture for BP.
The paired single-channel deepSimDEF architecture consists of 7 layers for functional similarity measurement of genes and gene products using their Gene Ontology term annotations from one of the GO sub-ontologies. For two input genes, their annotations are fed to the network in the first layer in which they will be represented as two lists of 100-dimensional embedding vectors. After applying dropout (0.15), max-pooling layer condenses each of these two lists into a 100-dimensional row-vector. Merge and highway layers together encode the degree of shared information of these two pooled vectors. In several locations of the architecture, fully-connected layers are considered for better representation of their underlying layers (followed by dropout of 0.3). Finally, based on the biological application in hand, the result of the prediction network is computed which can be either a scalar or a classification probability. The weights of the two pairs are shared during the network training and testing. This architecture is shown for BP, however, for CC and MF it stays the same with only having different length of the input annotations in the first layer that is defined by the maximum number of annotations given to a gene from that sub-ontology.

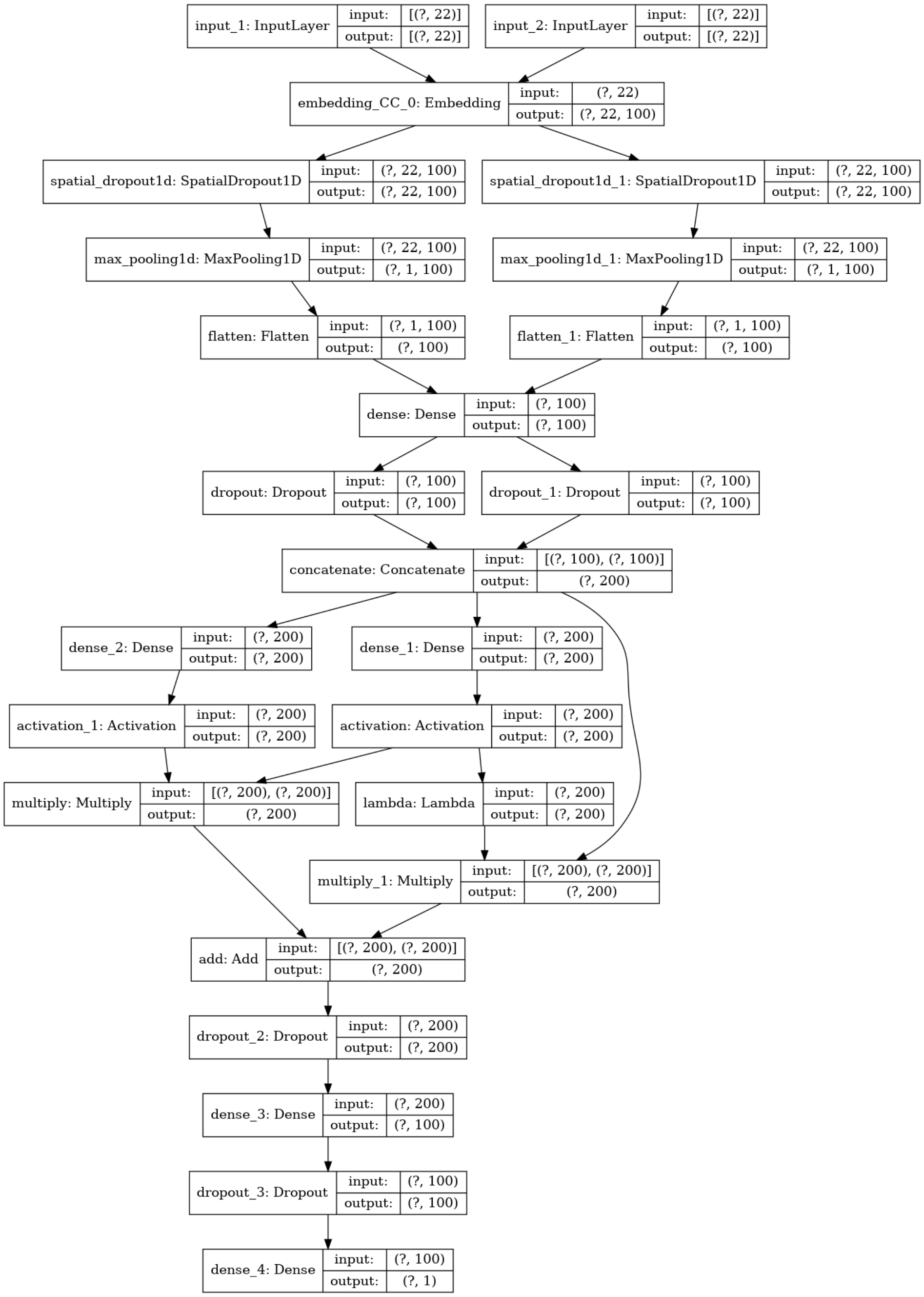

Figure 3 Paired single-channel deepSimDEF network architecture for CC.
The paired single-channel deepSimDEF architecture consists of 7 layers for functional similarity measurement of genes and gene products using their Gene Ontology term annotations from one of the GO sub-ontologies. For two input genes, their annotations are fed to the network in the first layer in which they will be represented as two lists of 100-dimensional embedding vectors. After applying dropout (0.15), max-pooling layer condenses each of these two lists into a 100-dimensional row-vector. Merge and highway layers together encode the degree of shared information of these two pooled vectors. In several locations of the architecture, fully-connected layers are considered for better representation of their underlying layers (followed by dropout of 0.3). Finally, based on the biological application in hand, the result of the prediction network is computed which can be either a scalar or a classification probability. The weights of the two pairs are shared during the network training and testing. This architecture is shown for CC, however, for BP and MF it stays the same with only having different length of the input annotations in the first layer that is defined by the maximum number of annotations given to a gene from that sub-ontology.

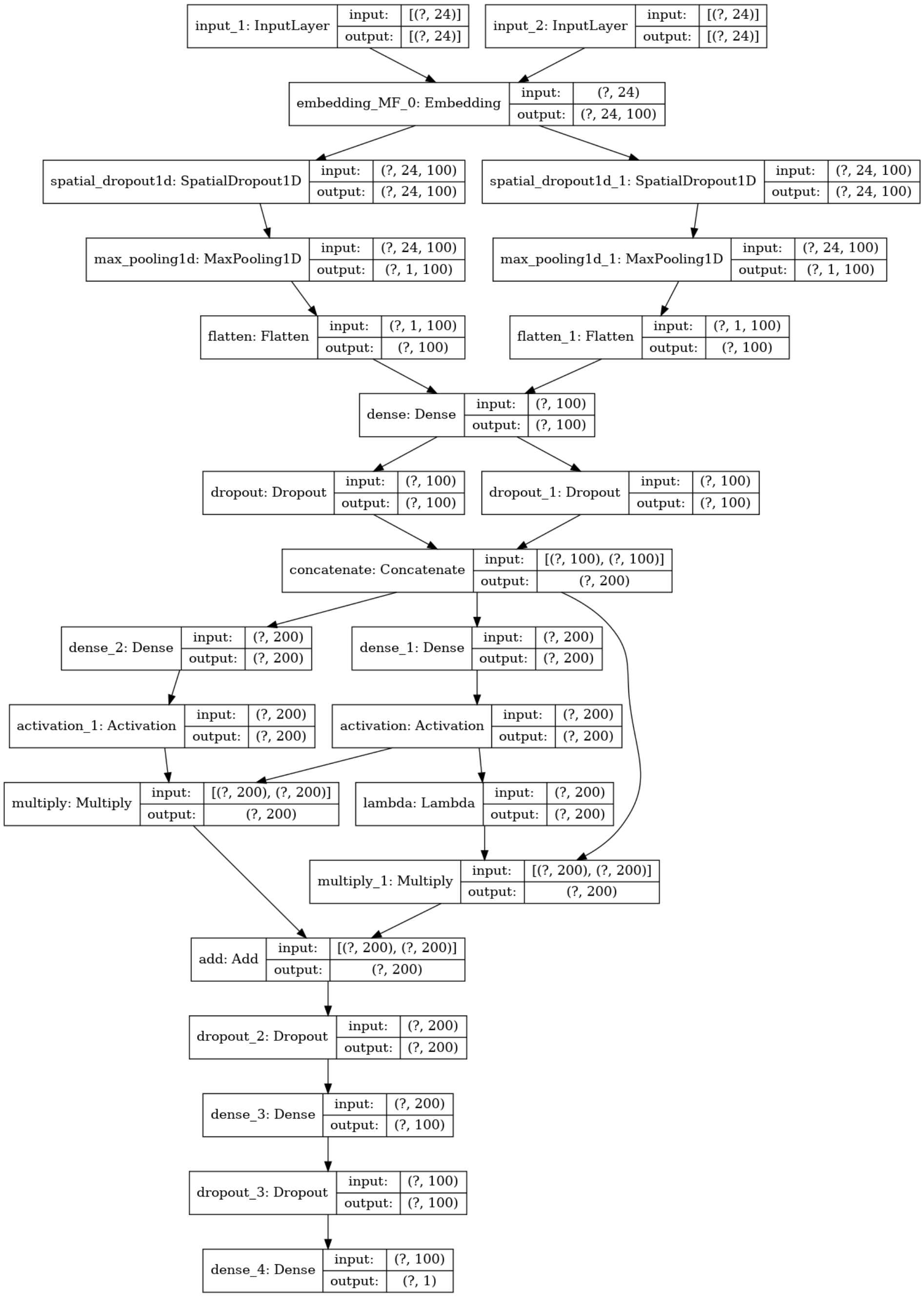

Figure 4 Paired single-channel deepSimDEF network architecture for MF.
The paired single-channel deepSimDEF architecture consists of 7 layers for functional similarity measurement of genes and gene products using their Gene Ontology term annotations from one of the GO sub-ontologies. For two input genes, their annotations are fed to the network in the first layer in which they will be represented as two lists of 100-dimensional embedding vectors. After applying dropout (0.15), max-pooling layer condenses each of these two lists into a 100-dimensional row-vector. Merge and highway layers together encode the degree of shared information of these two pooled vectors. In several locations of the architecture, fully-connected layers are considered for better representation of their underlying layers (followed by dropout of 0.3). Finally, based on the biological application in hand, the result of the prediction network is computed which can be either a scalar or a classification probability. The weights of the two pairs are shared during the network training and testing. This architecture is shown for MF, however, for CC and BP it stays the same with only having different length of the input annotations in the first layer that is defined by the maximum number of annotations given to a gene from that sub-ontology.
